# Metapopulation model of phage therapy of an acute *Pseudomonas aeruginosa* lung infection

**DOI:** 10.1128/msystems.00171-24

**Published:** 2024-09-04

**Authors:** Rogelio A. Rodriguez-Gonzalez, Quentin Balacheff, Laurent Debarbieux, Jacopo Marchi, Joshua S. Weitz

**Affiliations:** 1 Interdisciplinary Graduate Program in Quantitative Biosciences,Georgia Institute of Technology, Atlanta, Georgia, USA; 2 School of Biological Sciences, Georgia Institute of Technology, Atlanta, Georgia, USA; 3 CHU Félix Guyon, Service des maladies respiratoires, La Réunion, France; 4 Department of Microbiology, Institut Pasteur, Université Paris Cité, CNRS UMR6047, Bacteriophage Bacterium Host, Paris, France; 5 Department of Biology, University of Maryland, College Park, Maryland, USA; 6 Department of Physics, University of Maryland, College Park, Maryland, USA; 7 Institut de Biologie, École Normale Supérieure, Paris, France; Rice University, Houston, Texas, USA

**Keywords:** bacteriophage therapy, microbial ecology, mathematical modeling, infectious disease, lung infection, *Pseudomonas aeruginosa*, virology, antimicrobial agents, antibiotic resistance, innate immunity

## Abstract

Infections caused by multidrug resistant (MDR) pathogenic bacteria are a global health threat. Bacteriophages (“phage”) are increasingly used as alternative or last-resort therapeutics to treat patients infected by MDR bacteria. However, the therapeutic outcomes of phage therapy may be limited by the emergence of phage resistance during treatment and/or by physical constraints that impede phage–bacteria interactions *in vivo*. In this work, we evaluate the role of lung spatial structure on the efficacy of phage therapy for *Pseudomonas aeruginosa* infections. To do so, we developed a spatially structured metapopulation network model based on the geometry of the bronchial tree, including host innate immune responses and the emergence of phage-resistant bacterial mutants. We model the ecological interactions between bacteria, phage, and the host innate immune system at the airway (node) level. The model predicts the synergistic elimination of a *P. aeruginosa* infection due to the combined effects of phage and neutrophils, given the sufficient innate immune activity and efficient phage-induced lysis. The metapopulation model simulations also predict that MDR bacteria are cleared faster at distal nodes of the bronchial tree. Notably, image analysis of lung tissue time series from wild-type and lymphocyte-depleted mice revealed a concordant, statistically significant pattern: infection intensity cleared in the bottom before the top of the lungs. Overall, the combined use of simulations and image analysis of *in vivo* experiments further supports the use of phage therapy for treating acute lung infections caused by *P. aeruginosa,* while highlighting potential limits to therapy in a spatially structured environment given impaired innate immune responses and/or inefficient phage-induced lysis.

**IMPORTANCE:**

Phage therapy is increasingly employed as a compassionate treatment for severe infections caused by multidrug-resistant (MDR) bacteria. However, the mixed outcomes observed in larger clinical studies highlight a gap in understanding when phage therapy succeeds or fails. Previous research from our team, using *in vivo* experiments and single-compartment mathematical models, demonstrated the synergistic clearance of acute *P. aeruginosa* pneumonia by phage and neutrophils despite the emergence of phage-resistant bacteria. In fact, the lung environment is highly structured, prompting the question of whether immunophage synergy explains the curative treatment of *P. aeruginosa* when incorporating realistic physical connectivity. To address this, we developed a metapopulation network model mimicking the lung branching structure to assess phage therapy efficacy for MDR *P. aeruginosa* pneumonia. The model predicts the synergistic elimination of *P. aeruginosa* by phage and neutrophils but emphasizes potential challenges in spatially structured environments, suggesting that higher innate immune levels may be required for successful bacterial clearance. Model simulations reveal a spatial pattern in pathogen clearance where *P. aeruginosa* are cleared faster at distal nodes of the bronchial tree than in primary nodes. Interestingly, image analysis of infected mice reveals a concordant and statistically significant pattern: infection intensity clears in the bottom before the top of the lungs. The combined use of modeling and image analysis supports the application of phage therapy for acute *P. aeruginosa* pneumonia while emphasizing potential challenges to curative success in spatially structured *in vivo* environments, including impaired innate immune responses and reduced phage efficacy.

## INTRODUCTION

The antimicrobial resistance crisis poses a major burden to global health, with ~1.3 million deaths attributed to drug-resistant bacteria in 2019 alone (1). The discovery of new antibiotics has slowed (2), with only two new chemical classes of antibiotics approved since 2017. The antimicrobial resistance crisis has also catalyzed the search for alternative therapies, including phage therapy. Phage therapy conventionally uses bacteriophage (phage) to target and kill specific bacterial pathogens. However, the emergence of phage resistance and/or physical constraints hindering phage propagation can limit phage treatment efficacy.

The ability of phage to kill bacteria is often assessed through *in vitro*, liquid medium assays. In these well-mixed systems, phage can efficiently eliminate both Gram-positive (3) and Gram-negative (3, 4) bacterial pathogens. In contrast, phages are less efficient at clearing bacterial populations with complex, spatial structure (5–8), especially well-established and mature biofilms (5, 7, 8). The spatial heterogeneity of biofilms limits phage propagation and biofilm clearance (9, 10). For instance, slow-growing bacteria living at the center of a biofilm restrict phage propagation and impede biofilm elimination (9). Furthermore, clusters of phage-resistant bacteria can protect susceptible bacteria by blocking phage infection in mixed-strain biofilms (10).

Applications of phage therapy *in vivo* and in the clinic require consideration of the context, including the spatial structure and host immune responses. For example, a three-phage cocktail targeting *Escherichia coli* in the murine gut resulted in the coexistence of phage and bacteria (11). This study also found a significantly higher relative abundance of phage in the luminal part of the ileum than in the mucosal part (11), suggesting that the intestinal mucosa serves as a spatial refuge for bacteria. Further supporting the spatial refuge hypothesis, an independent study found that high T7 phage and *E. coli* titers coexist in the mice colon for up to 3 weeks, with 80% of isolated *E. coli* colonies susceptible to parental phage (12). The host immune responses also play a significant role in shaping phage therapy outcomes *in vivo*. For instance, synergistic interactions between phage and neutrophils enabled clearance of *P. aeruginosa* lung infections in WT mice (13). In contrast, phage therapy failed to eradicate *P. aeruginosa* infection in neutropenic mice (13, 14). Moreover, phage immunogenicity assays indicate that phages do not significantly increase cytokine production and are often well-tolerated by mammalian hosts (13, 15). However, prolonged treatment (~6 months) with intravenously administered phage might induce neutralizing antibody responses against phage (16).

Computational models and theory provide a route to evaluate the impacts of immune responses on the potential efficacy of phage therapy. For example, influential modeling work integrating immune mechanisms into phage therapy (17, 18) assessed the combined effects of phage life history traits and immune response intensity on bacterial control. These models assumed that the immune response could grow unconstrained and that bacteria cannot evade immune responses. As a result, these models predicted scenarios where the host immune system alone inevitably controls the bacterial infection (17, 18). Recent work incorporating more nuanced models revealed how phage and a sufficiently effective innate immune system work synergistically to clear infections (13, 19). Such “immunophage synergy” may also apply to phage–antibiotic combination therapy (20). It is important to note that these models (13, 17–19) do not explicitly represent spatial dynamics, a critical component when modeling phage therapy *in vivo*.

Spatially explicit models can be used to assess infection dynamics and therapeutic outcomes *in vivo* (21). For instance, spatial structure limits phage dispersion and enables spatial refuges for bacteria to survive phage infection, facilitating the coexistence of phage and bacteria (22, 23). In the context of phage–antibiotic combination therapy, an individual-based model (IBM) found that double-resistant mutants emerged when phage dispersion was restricted and antibiotics were heterogeneously distributed. This led to drug-free spatial refuges where bacteria replicate and acquire phage- and antibiotic-resistant mutations (22). Moreover, an IBM of phage treatment of a mixed-strain biofilm identified that fitness costs associated with phage resistance, coupled with spatial protection of susceptible cells vs phage infection by clusters of resistant bacteria, facilitated the long-term coexistence of susceptible and resistant strains (10).

Spatial organization into subpopulations has been proposed as a key factor in fostering the persistence of predator–prey interactions (24). Metapopulation models represent environments as interconnected patches where local subpopulations reside and interact via individuals moving among the patches. A metapopulation model of the host–parasitoid pair, *Callosobruchus chinensis* and *Anisopteromalus clalandrae*, found that the persistence of the host–parasitoid interaction increased with spatial subdivision and with the number of patches (24). In a host plant–pathogen metapopulation model, limited host dispersion led to increased pathogen diversity, while increased host dispersion homogenized the dynamics of local populations and decreased pathogen diversity (25). Additionally, a metapopulation model exploring pathogen circulation in healthcare settings found that pathogen control via host sanitation, rather than environment sanitation, led to more rapid elimination of the circulating pathogen (26).

In this study, we introduce a metapopulation model that integrates lung spatial structure and host immune responses to evaluate their combined impact on phage therapy outcomes for acute pneumonia caused by *P. aeruginosa*. The model simulates ecological interactions between two bacterial strains (phage-susceptible and phage-resistant), phage, and the innate immune response within a network structure resembling the branching pattern of the lungs. Our findings reveal that the clearance of a *P. aeruginosa* infection is contingent upon sufficiently active innate immune states and suitable phage life history traits. Moreover, we note that the spatial model requires higher innate immune response levels to increase the chances of therapeutic success, in contrast to a well-mixed model, which predicts infection resolution with lower innate immune levels, highlighting the spatial structure’s role in shaping phage therapy outcomes. Throughout our simulations, we observe a spatial pattern wherein infection clears faster at the network’s bottom compared to the top, demonstrating robustness across varied distributions of the bacterial inoculum and phage dose. Lastly, our analysis of *in vivo* mice lung infection data (13) identifies a concordant and statistically significant pattern: a bottom-to-top spatial transition in pathogen clearance, aligning with the predictions of the metapopulation model.

### Model

**Fig 1:**
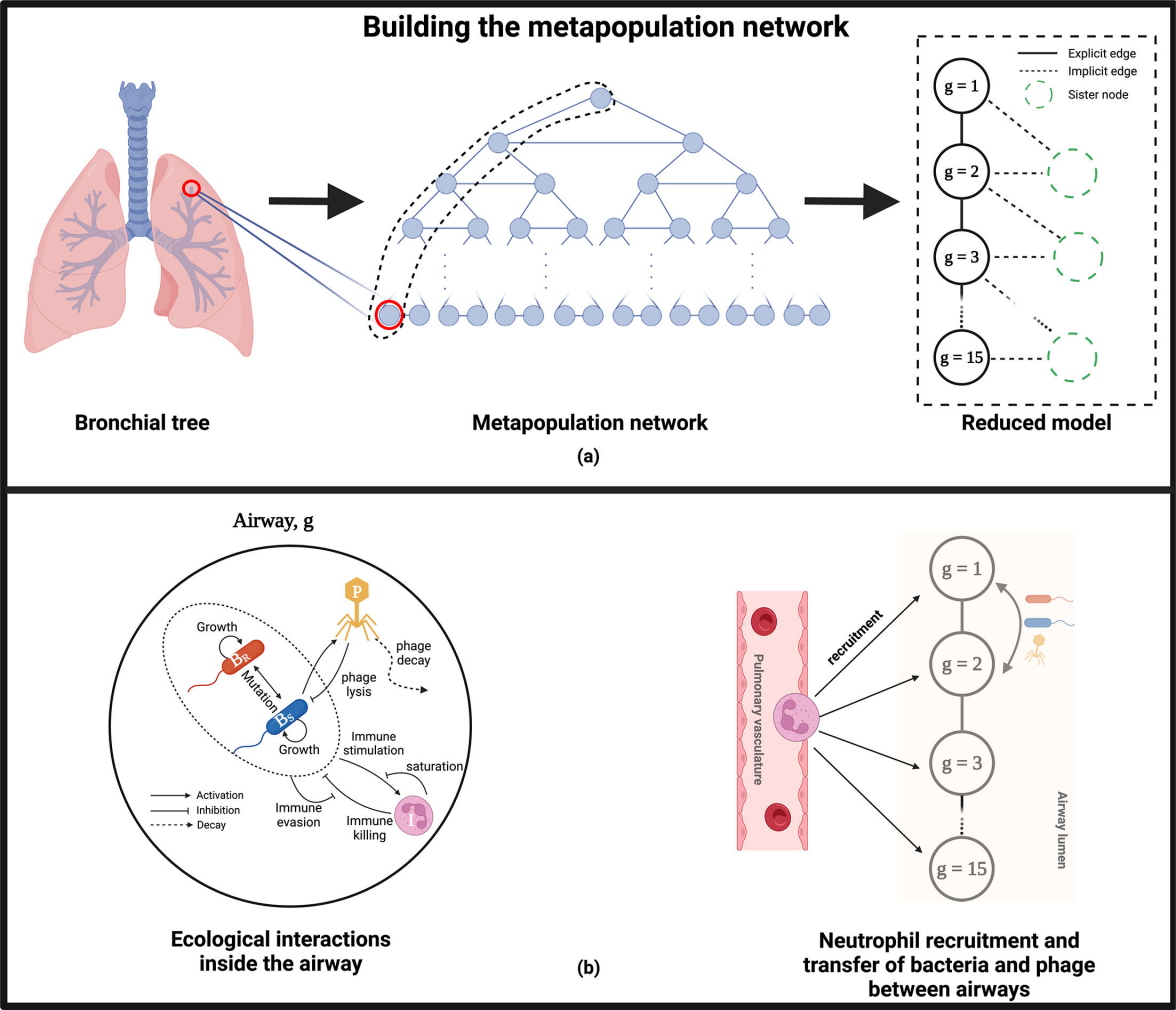
Schematic of the metapopulation network model of phage therapy of a *P. aeruginosa* infection. The structure of the metapopulation network is based on the geometry of a symmetrical bronchial tree with a dichotomous branching pattern (**a**). The airways of the bronchial tree (left) are represented by the network nodes (a-middle), while the network links represent the branching points of the bronchial tree. For example, the section that connects the trachea to the left and right main bronchi is considered a branching point. We assume that the bronchial tree is symmetrical such that the left and right parts of the tree are identical and so are their dynamics in a deterministic system. Therefore, we only focus on one side of the tree (the dashed line on the middle network) and reduce the number of nodes in our original network to one node per generation. The final network topology of this reduced model consists of 15 connected nodes that form a chain (right). The degree of the network nodes is 4, except for the first and last nodes, which have a degree of 2. In panel **b**, we show the ecological interactions between phage-susceptible bacteria ($B_{S}$), phage-resistant bacteria ($B_{R}$), phage (P), and the host innate immune response (I) at the node level (left). Phage infects the susceptible strain, while the resistant strain is targeted only by the innate immune response. The immune response grows in the presence of bacteria and targets both bacterial strains. Neutrophils are recruited to the site of the infection from the pulmonary vasculature, while phage and bacteria transfer between connected nodes to spread across the network (right).

The metapopulation network structure is based on the geometry of a symmetrical bronchial tree with a dichotomous branching pattern (Fig. 1a). The nodes of the network represent the airways, and the network links represent the branching points on the bronchial tree. We assume bacteria colonize the airways (nodes) and spread across the network through the network links. We consider the structure of the bronchial tree to be perfectly symmetrical, such that the left and right sides of the tree are identical. Hence, in the absence of stochastic effects, the dynamics at every node of the same tree generation, g, will be identical. Therefore, we consider only one node in each generation without losing information on the whole network. The final network topology consists of a chain of connected nodes (Fig. 1a-right), where each node represents one airway in each generation (g) of the tree. From the second generation, each level comprises a sister node identical to node g, which is topologically connected to g and g−1.

To determine the number of generations of the metapopulation network, we estimate the volume of the network in terms of the ~1 mL total lung capacity (TLC) of mice (27). To do so, we calculate the volume of individual airways of different generations using the anatomical airway information (28). Then, we consider the number of airways per generation (i.e., $2^{g-1}$ airways per generation, g) to estimate the network volume. We estimated that 15 generations yield a network volume of 0.9 mL, which does not exceed the mouse TLC.

Nonlinear interactions between bacteria, phage, and neutrophils govern the population dynamics at the node level, corresponding to a single airway at generation g, Fig. 1b-left. By using a system of nonlinear, ordinary differential equations (equations 1–4), we represent the ecological interactions among bacteria, phage, and host innate immunity occurring at the node level (g). We propose a model with two *P. aeruginosa* strains, one of which is phage-susceptible ($B_{S}$) and a second which is phage-resistant ($B_{R}$). The growth of bacteria is density-dependent and is limited by the carrying capacity, $K_{C}$ can be infected and lysed by phage (P), while $B_{R}$ resists phage infection. Phage, P, replicates inside bacteria and decay in the environment at a rate ω. Neutrophils target and kill both $B_{S}$ and $B_{R}$ bacterial strains. The dynamics are as follows:


(1) $$\begin{array}{l} \frac{dB_{S,g}}{dt}=\overset{B_{S}\text{ Growth}}{\overbrace{r_{P}B_{S,g}\left(1-\frac{B_{tot,g}}{K_{C}}\right)}}\overset{\text{Mutation to }B_{R}}{\overbrace{\left(1-\mu_{1}\right)}}+\overset{\text{Mutation from }B_{R}}{\overbrace{\mu_{2}r_{A}B_{R,g}\left(1-\frac{B_{tot,g}}{K_{C}}\right)}}\\ \;-\overset{\text{Immune killing}}{\overbrace{\frac{\varepsilon I_{g}B_{S,g}}{1+\frac{B_{tot,g}}{K_{D}}}}}-\overset{\text{Lysis}}{\overbrace{B_{S,g}F(P_{g})}}-\overset{\text{Outflux}}{\overbrace{\frac{8D_{B}}{{l^{2}}_{g}}B_{S,g}}}+\overset{\text{Influx from g-1}}{\overbrace{\left(\frac{8D_{B}}{{l^{2}}_{g-1}}\rho_{g-1}B_{S,g-1}\frac{V_{g-1}}{V_{g}}\right)(1-\delta_{1,g})}}\\ \;+\overset{\text{Influx from g (sister branch)}}{\overbrace{\left(\frac{8D_{B}}{{l^{2}}_{g}}\rho_{g}B_{S,g}\right)(1-\delta_{1,g})}}+\overset{\text{Influx from g + 1}}{\overbrace{\left(2\frac{8D_{B}}{{l^{2}}_{g+1}}\rho_{g+1}B_{S,g+1}\frac{V_{g+1}}{V_{g}}\right)(1-\delta_{N,g})}} \end{array},$$

(2) $$\begin{array}{l} \frac{dB_{R,g}}{dt}=\overset{B_{R}\text{ Growth}}{\overbrace{r_{A}B_{R,g}\left(1-\frac{B_{tot,g}}{K_{C}}\right)}}\overset{\text{Mutation to }B_{S}}{\overbrace{\left(1-\mu_{2}\right)}}+\overset{\text{Mutation from }B_{S}}{\overbrace{\mu_{1}r_{P}B_{S,g}\left(1-\frac{B_{tot,g}}{K_{C}}\right)}}\\ \;\;-\overset{\text{Immune killing}}{\overbrace{\frac{\varepsilon I_{g}B_{R,g}}{1+\frac{B_{tot,g}}{K_{D}}}}}-\overset{\text{Outflux}}{\overbrace{\frac{8D_{B}}{{l^{2}}_{g}}B_{R,g}}}+\overset{\text{Influx from g-1}}{\overbrace{\left(\frac{8D_{B}}{{l^{2}}_{g-1}}\rho_{g-1}B_{R,g-1}\frac{V_{g-1}}{V_{g}}\right)(1-\delta_{1,g})}}\\ \;\;+\overset{\text{Influx from g (sister branch)}}{\overbrace{\left(\frac{8D_{B}}{{l^{2}}_{g}}\rho_{g}B_{R,g}\right)(1-\delta_{1,g})}}+\overset{\text{Influx from g + 1}}{\overbrace{\left(2\frac{8D_{B}}{{l^{2}}_{g+1}}\rho_{g+1}B_{R,g+1}\frac{V_{g+1}}{V_{g}}\right)(1-\delta_{N,g})}} \end{array},$$

(3) $$\begin{array}{l} \frac{dP_{g}}{dt}=\overset{\text{Viral release}}{\overbrace{\beta B_{S,g}F(P_{g})}}-\overset{\text{Decay}}{\overbrace{\omega P_{g}}}-\overset{\text{Outflux}}{\overbrace{\frac{8D_{P}}{{l^{2}}_{g}}P_{g}}}+\overset{\text{Influx from g-1}}{\overbrace{\left(\frac{8D_{P}}{{l^{2}}_{g-1}}\rho_{g-1}P_{g-1}\frac{V_{g-1}}{V_{g}}\right)(1-\delta_{1,g})}}\\ +\overset{\text{Influx from g (sister branch)}}{\overbrace{\left(\frac{8D_{P}}{{l^{2}}_{g}}\rho_{g}P_{g}\right)(1-\delta_{1,g})}}+\overset{\text{Influx from g + 1}}{\overbrace{\left(2\frac{8D_{P}}{{l^{2}}_{g+1}}\rho_{g+1}P_{g+1}\frac{V_{g+1}}{V_{g}}\right)(1-\delta_{N,g})}} \end{array},$$

(4) $$\begin{array}{l} \frac{dI_{g}}{dt}=\overset{\text{Immune stimulation}}{\overbrace{\alpha I_{g}\left(1-\frac{I_{g}}{K_{I}}\right)\left(\frac{B_{tot,g}}{B_{tot,g}+K_{N}}\right)\left(1-\frac{\sum_{g=1}^{15}I_{g}V_{g}2^{g-1}}{N_{Lung}}\right)}} \end{array},$$

In this model, $B_{S}$ grows at a maximum rate $r_{P}$, while $B_{R}$ grows at a maximum rate $r_{A}$, and the total bacterial density is represented by $B_{tot}=B_{S}+B_{R}$. Phages infect and lyse the phage-susceptible bacteria ($B_{S}$) at a rate F(P) with a burst size of β. The host innate immune response (I) is activated in the presence of bacteria and grows at a maximum rate α, while $K_{N}$ represents the bacterial density at which the growth of the immune response is half-saturated (29).

We model a saturating immune response by including a local density carrying capacity in the immune growth, $K_{I}$, as it was shown in previous experimental works that the innate immune response cannot grow unconstrained due to a finite number of immune cells that can be produced and tolerated (30, 31). Moreover, we use the term *N*_Lung_, representing the observed neutrophil count in the mice lungs (31), to globally constrain neutrophil recruitment across the bronchial network. The innate immune response kills both susceptible and resistant bacteria with a maximum killing rate ε. However, bacteria at high densities can activate mechanisms to evade the immune response, such as biofilm formation (32) and the production of virulence factors (33), resulting in reduced immune killing efficiency. We account for this by introducing the saturation at high bacterial load of the immune killing rate (i.e., we scale the immune killing term by the denominator $1+B_{tot}/K_{D}$), in agreement with the experimental measurements of *in vivo* bacteria burden (34, 35), where $K_{D}$ is the bacterial density at which the immune killing rate is half its maximum.

These local reaction terms in each node in our metapopulation model build on previous works that established an “immunophage synergy” regime (13, 19) in which both phage and the host innate immune response are required to eliminate the bacterial infection; i.e., the infection persists when only one or neither is present. The immunophage synergy regime has been previously characterized mathematically (19), where immune saturation and bacterial immune evasion were identified as key ingredients leading to such a regime. Immunophage synergy was then observed *in vivo* by infecting mice with varying immune statuses with pathogenic bacteria and treating them with phage (13).

Bacteria and phage can hop between connected nodes (Fig. 1b-right). The rate at which they hop is determined by the time, $\tau_{g}$, required for each species to cross half of the airway length ($l_{g}$) via diffusion D. The hopping rate is calculated as the reciprocal of these times, $\frac{1}{\tau_{g}}$. In the model, influx and outflux terms govern the transfer of bacteria and phage between neighboring nodes. We assume a homogeneous spreading between airways such that the links between nodes are non-weighted and the flux from node j to node g is inversely proportional to the degree of node j (i.e., $\rho_{j}=1/d_{j}$). Additionally, the number of bacteria and phage transferred from a neighbor node j to a local node g is rescaled to the local density, i.e., $\frac{B_{S,j}V_{j}}{V_{g}}$. Note that we multiply by 2 the influx terms from g+1 into g since node g is linked to both the main node g+1 and its sister node in the reduced network topology (Fig. 1a-right), although we do not need to explicitly model the dynamics in sister nodes by virtue of the branching tree symmetry.

Changes in the concentration of mucin lining the airways impact bacterial motility (36) and phage diffusion (37, 38), thereby affecting their hopping rate throughout the bronchial network. Text S1 B-C explains in detail how to calculate the diffusion constants and the hopping rate of phage and bacteria across different mucin levels. Additionally, the diffusion values of phage and bacteria can be found in Table S1.

For phage infection, we use a model, F(P), that assumes that spatial heterogeneities inside the mouse lungs might limit phage–bacteria encounters. $F(P)=\tilde{\phi }P^{\sigma }$, where $\tilde{\phi }$ is the nonlinear phage adsorption rate and σ<1 is the power-law exponent in the phage infection rate. This heterogeneous mixing model (13) has been used previously to recapitulate phage–bacteria dynamics *in vivo*.

The parameter values used in model simulations, largely chosen leveraging the inference work done in (13), are listed in Table 1. Additional details on the model, network structure, calculation of bacteria and phage transfer rates, and statistical analysis are provided in Text S1.

**TABLE 1:** Parameter values of the metapopulation network model. Concentrations are calculated based on the mice lung volume of 0.9 mL

Parameters of the metapopulation model	Value	Estimated from
$r_{P}$, maximum growth rate of phage-susceptible bacteria	0.75 h^−1^	*P. aeruginosa* murine pneumonia model (35)
$r_{A}$, maximum growth rate of phage-resistant bacteria	0.675 h^−1^	10% tradeoff between resistance against the phage and growth rate (39)
$K_{C}$, carrying capacity of bacteria	1.49 × 10^9^ CFU/mL	Assuming ~4 times above the typical bacterial density in wild-type mice 24 hours post-infection, estimated from reference 13
β, burst size of phage	100	Estimated from reference 13
ω, decay rate of phage	0.07 h^−1^	Estimated from reference 13
ε, killing rate parameter of the immune response	5.47 × 10^7^ mL/(h cell)	Set such that $\varepsilon K_{I}$ gives the maximum granulocyte killing rate (35)
α, maximum growth rate of the immune response	0.07 h^−1^	Fitting of neutrophil recruitment data (31)
$K_{I}$, maximum capacity of the immune response	3.59 × 10^6^ cell/mL	Fitting of neutrophil recruitment data (31)
$K_{D}$, bacteria concentration at which the immune response is half as effective	6.14 × 10^6^ CFU/mL	Corresponds to a lethal dose of about 5.5 × 10^6^ CFU/lung
$K_{N}$, bacteria concentration when the immune response growth rate is half its maximum	10^7^ CFU/mL	*In vitro* data of TLR5 response to PAK strain (29)
$B_{0}$, initial bacterial density (when the inoculum is uniformly distributed among network nodes)	1.1106 × 10^6^ CFU/mL	Total inoculum of 10^6^ CFU
$P_{0}$, initial phage dose (when the dose is uniformly distributed among network nodes)	1.1106 × 10^7^ PFU/mL	Total phage dose of 10^7^ PFU
$I_{0}$, initial immune response	4.048 × 10^5^ cell/mL	Fitting of neutrophil recruitment data (31)
$I_{0}$, initial immune response (neutropenic mice)	0 cell/mL	Assuming no primary innate immunity
$N_{Lung}$, total number of neutrophils in the lungs	3.24 × 10^6^ cells	Fitting of neutrophil recruitment data (31)
$\mu_{1}$, probability of emergence of phage-resistant mutants per cellular division	2.85 × 10^−8^	Estimated from experimental measurements (40)
$\mu_{2}$, probability of emergence of reversible mutants (i.e., from phage-resistant to phage-susceptible) per cellular division	2.85 × 10^−8^	Approximated to the estimates from reference 40

Heterogeneous mixing (HM) model parameters		
$\tilde{\phi }$, nonlinear phage adsorption rate	$1.686\times 10^{-7}$ ${\left(\text{mL/PFU}\right)}^{\sigma }$ ${\text{h}}^{-1}$	Estimated from reference 13
σ, power-law exponent in the phage infection rate	0.6	Estimated from reference 13

Anatomical information of airways	Range	
$V_{g}$, airway volume	4.8 × 10^−3^ to 2.2 × 10^−5^ mL	Calculated as the volume of a cylinder ($\pi r^{2}h$), using anatomical information of mice airways from reference 28
$l_{g}$, airway length	0.76–0.06 cm	Anatomical information of mice airways from reference 28
Airway diameter	0.089–0.02 cm	Anatomical information of mice airways from reference 28

## RESULTS

### Spatiotemporal dynamics of phage therapy of acute pneumonia caused by *P. aeruginosa*

**Fig 2:**
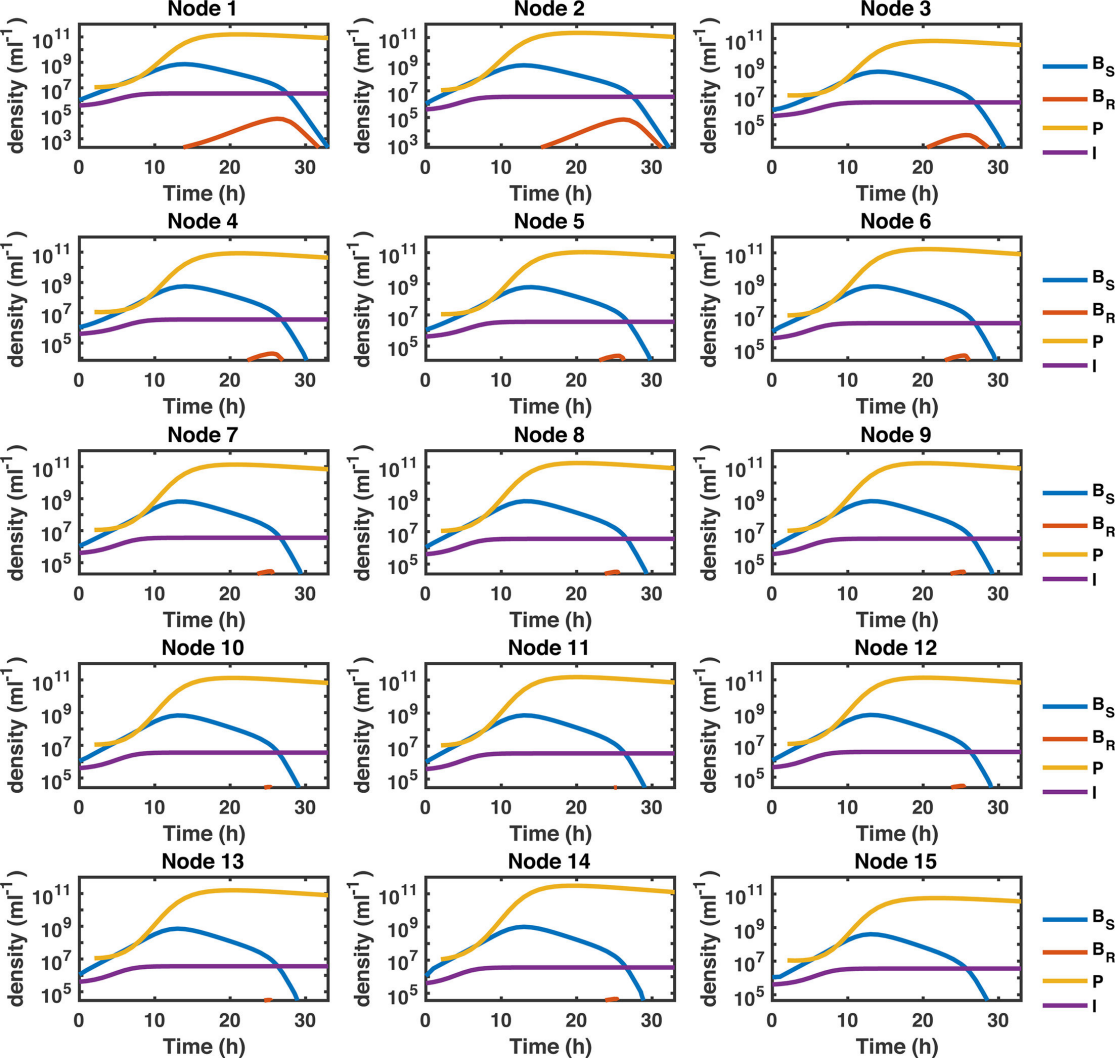
Spatiotemporal dynamics of phage therapy of a *P. aeruginosa* lung infection. Population dynamics at the node level for phage (solid yellow line), phage-susceptible bacteria (solid blue line), phage-resistant bacteria (solid orange line), and the host innate immune response (purple solid line). We set the lower bound of the *y*-axis for each node panel to the $1/V_{i}$ density level, such that only densities corresponding to species numbers >1 CFU (PFU or cell) are depicted, where $V_{i}$ is the volume of node i. Simulation configuration is provided in the main text with parameter values in Table 1 and computational details in Text S1.

We begin by simulating phage therapy treatment of a *P. aeruginosa* infection in an immunocompetent host. The infection starts by inoculating the host with $10^{6}$ bacterial cells. For phage treatment, we add $10^{7}$ phage 2 hours after the beginning of the infection. The bacterial inoculum and the phage dose are uniformly distributed among network nodes, ensuring that each node has the same initial bacterial density of $1.11\times 10^{6}$ CFU/mL and phage density of $1.11\times 10^{7}$ PFU/mL, considering a network volume of ∼0.9 mL. We simulate baseline neutrophil levels by setting an initial immune density of $4.05\times 10^{5}$ neutrophils/mL in all network nodes (31). Finally, considering that we model phage treatment dynamics in realistic physical compartments with actual volume differences, we show in Fig. 2 the population dynamics (in densities) of bacteria, phage, and the innate immune response at the node level when their numbers exceed 1 CFU (PFU or cell). Densities below this threshold are not shown.

By analyzing the dynamics that emerge from the phage therapy of a *P. aeruginosa* infection (Fig. 2), we observed that the phage-susceptible bacterial population ($B_{S}$) initially increased and reached its peak density after 13 hours. The phage population (P) grew together with the $B_{S}$ population during the first hours of the simulation. The host innate immune response (I) also increased in the presence of bacteria during the first 10 hours of the simulation. Once the phage population reached high-density levels, i.e., ∼$10^{11}$ PFU/mL, we observed a reduction in the $B_{S}$ population across the network. The bacterial elimination rate accelerated as the phage reduced the $B_{S}$ population density to a level that facilitated the innate immune response to control the infection. Although phage-resistant mutants ($B_{R}$) dynamics led to transient increase above $10^{4}$/mL in the top nodes of the network in contrast to bottom nodes (due to threshold effects mentioned in the previous paragraph), the $B_{R}$ population remained at levels that could be controlled by the host innate immune response. The combined effects of phage and neutrophils led to the clearance of the infection in the network after 32 hours (Fig. 2), similar to observed clearance times of 24–48 hours in phage treatment of *P. aeruginosa* in immunocompetent mice (13). Note that despite similar population dynamics between nodes, the infection clearance did not occur simultaneously in all the nodes. The infection resolved first in the bottom (∼28 hours) and later in the top nodes (∼33 hours).

### Joint action of phage and host innate immunity in bacterial infections

Next, we study how different treatment scenarios impact the bacterial infection spatiotemporal dynamics across the metapopulation network. We test four treatment scenarios that result from the presence (+) or absence (−) of both phage and innate immunity. As before, we inoculate a total number of 10^6^ bacterial cells that are homogeneously distributed in the network. When phage therapy is applied, we inoculate the host with 10^7^ phage (MOI ≈10) 2 hours after the beginning of the infection. If the host is immunocompetent, we set an initial immune density of $4.05\times 10^{5}$ neutrophils/mL for all network nodes, totaling $3.64\times 10^{5}$ neutrophils in the lungs (31). When the host is immunodeficient, we set the immune density to I=0 neutrophils/mL in the network nodes. The bacterial inoculum and the phage dose are uniformly distributed in the network such that each node has the same initial bacterial density ($1.11\times 10^{6}$ CFU/mL) and phage density ($1.11\times 10^{7}$ PFU/mL), as in the prior section.

**Fig 3:**
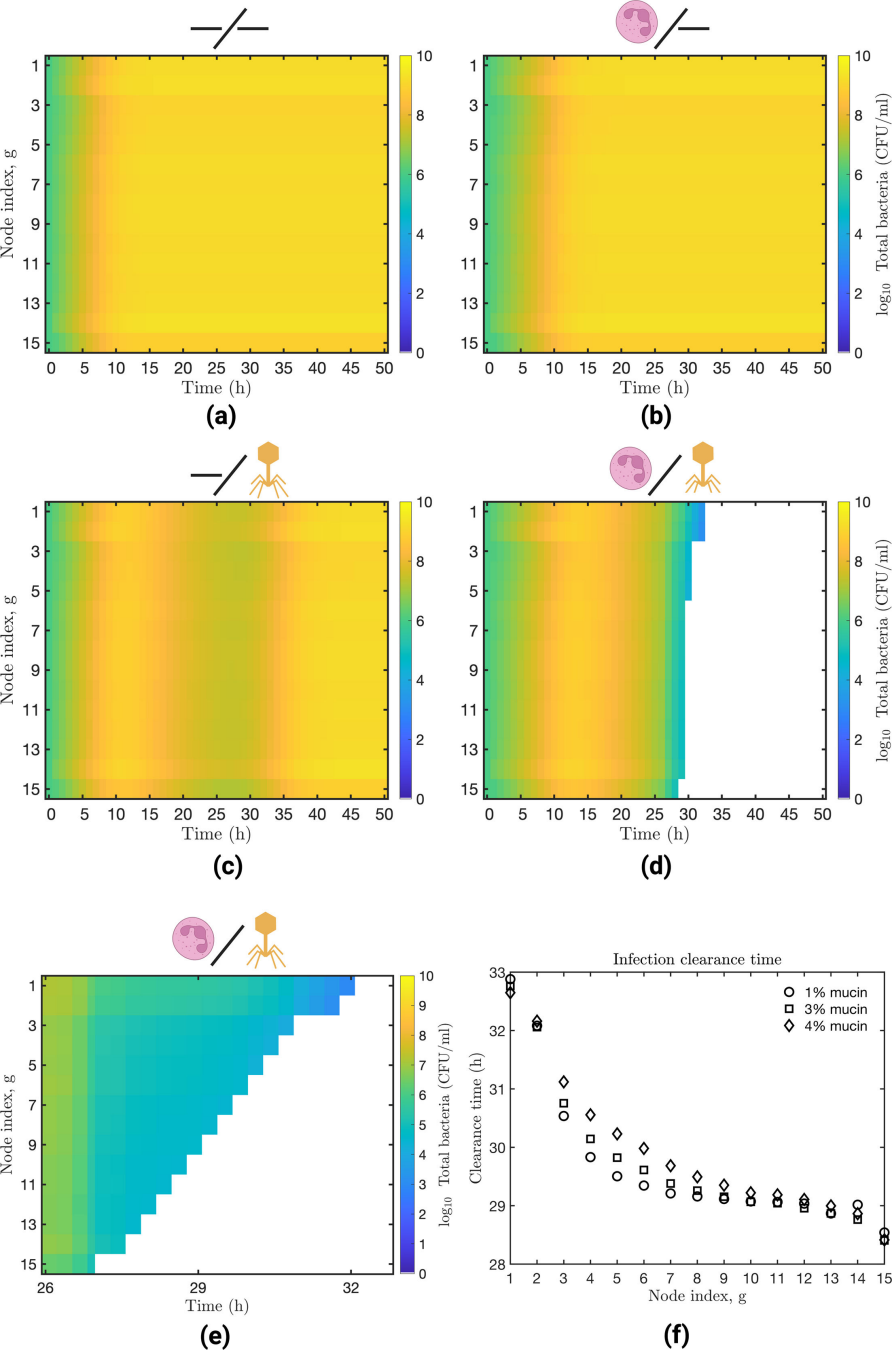
Bacterial dynamics under different phage and innate immune treatments. We simulate four treatment scenarios that result from the presence or absence of both phage and the innate immune response. We show the bacterial dynamics across the metapopulation network when the host is immunodeficient untreated (**a**) or phage-treated (**c**). Similarly, we show the bacterial dynamics when the host is immunocompetent untreated (**b**) or phage-treated (**d**). The heatmaps depict the progression of the bacterial infection across the network; each row represents a network node, g, while the columns indicate the simulation time (hour). The node color represents the bacterial density at a given time. The yellow regions represent high bacterial density, and the white areas represent infection clearance. When the host is immunocompetent and phage-treated, we zoom in and show the infection clearance pattern (**e**). Effects of varying the mucin level (1%–4%) on the infection clearance time (**f**). On the heatmaps, row 1 represents Generation 1 and the top of the lungs, while row 15 represents Generation 15 and the bottom of the lungs. The simulation configuration is provided in the main text with parameter values in Table 1 and computational details in Text S1.

When an immunodeficient host was not treated with phage, bacteria grew unimpeded (Fig. S2a), reaching carrying capacity levels ($1.5\times 10^{9}$ CFU/mL) after 10 hours in all the nodes (Fig. 3a). On the other hand, an active innate immune response delayed the initial growth of bacteria due to neutrophils killing the bacteria (Fig. 3b). However, in the absence of another antimicrobial effect, bacteria continued to grow and overwhelmed the innate immune response, rendering it insufficient for controlling the infection (Fig. S2b). The phage treatment of *P. aeruginosa* infection in an immunodeficient host led to the elimination of the $B_{S}$ population after 50 hours in most network nodes. However, it took about 90 hours to eliminate the $B_{S}$ population in the top nodes (Fig. S2c). During this time, phage-resistant mutants ($B_{R}$) emerged and spread throughout the network, causing the infection to persist (Fig. 3c). Finally, the model predicted therapeutic success when host innate immunity complemented phage therapy (Fig. 3d). We found this outcome to be robust to variations of up to 20% in the critical parameter $K_{D}$ (Fig. S3a) and to higher initial bacterial ($10^{7}$ CFU) and phage ($10^{8}$ PFU) inocula (Fig. S4), which are conditions consistent with *in vivo* treatment of the *P. aeruginosa* strain PAKlumi with the phage PAK_P1 (13). Model simulations indicated that bacteria are eliminated from the network after 32 hours. The elimination of the infection followed a spatial pattern that started at the bottom and continued to the top of the network (Fig. 3e); this pattern remained robust to variations in the parameter $K_{D}$ (Fig. S3b).

The spatial pattern of infection clearance arises in part due to volume differences among network nodes, with the bottom nodes being smaller (and having smaller volumes) than the top nodes. Although our model is based on densities, we define a bacterial extinction threshold based on cell counts in a node. Bacterial elimination at the node level occurs when the number of bacterial cells in a node (airway) falls below the 1 CFU threshold. Therefore, if the bottom nodes have smaller volumes than the top nodes, we expect fewer bacterial cells in the bottom nodes than in the top ones so that the extinction threshold would be reached faster at the bottom of the network. The speed at which the extinction threshold is reached depends on bacteria falling below a critical level ($K_{D}$), after which the immune system removes bacteria at a fixed rate ($\varepsilon K_{I}$) until complete elimination within each node. In Text S1 D, we further explain the factors giving rise to the spatial pattern of infection elimination and derive an approximation for the time to infection clearance at the node level.

Next, we sought to characterize how changes in mucin levels—known for shaping phage and bacteria propagation rates (36–38)—impact the observed clearance pattern. The spatial clearance pattern was recapitulated for mucin levels varying from 1% to 4% (Fig. 3f). This suggests infection clearance is robust to changes in conditions that affect phage and bacterial propagation rates when the host is fully immunocompetent. Overall, modeling outcomes show that synergistic interactions between phage and neutrophils may lead to the resolution of the infection in spatially structured environments.

### Varying the initial distribution of bacteria and phage in the metapopulation network

Thus far, we have explored the impacts of phage therapy on a *P. aeruginosa* infection model by uniformly distributing the bacterial inoculum and the phage dose in the network. However, the actual distribution of phage and bacteria in the bronchial tree after intranasal inoculation is not necessarily well-constrained. Hence, we try different forms of allocating the bacteria and phage inocula in the metapopulation network. For example, (i) we uniformly distribute the bacterial inoculum such that every node has the same initial density (uniform distribution), (ii) we distribute the inoculum among the first three nodes of the network (top distribution), (iii) we distribute the inoculum among the last twelve nodes of the network (bottom distribution), and (iv) we only inoculate the first node of the network (i.e., intratracheal instillation). We use the same distribution forms as the bacterial inoculum for the phage dose. In total, we test 16 ways of distributing the bacterial inoculum and the phage dose in the network.

**Fig 4:**
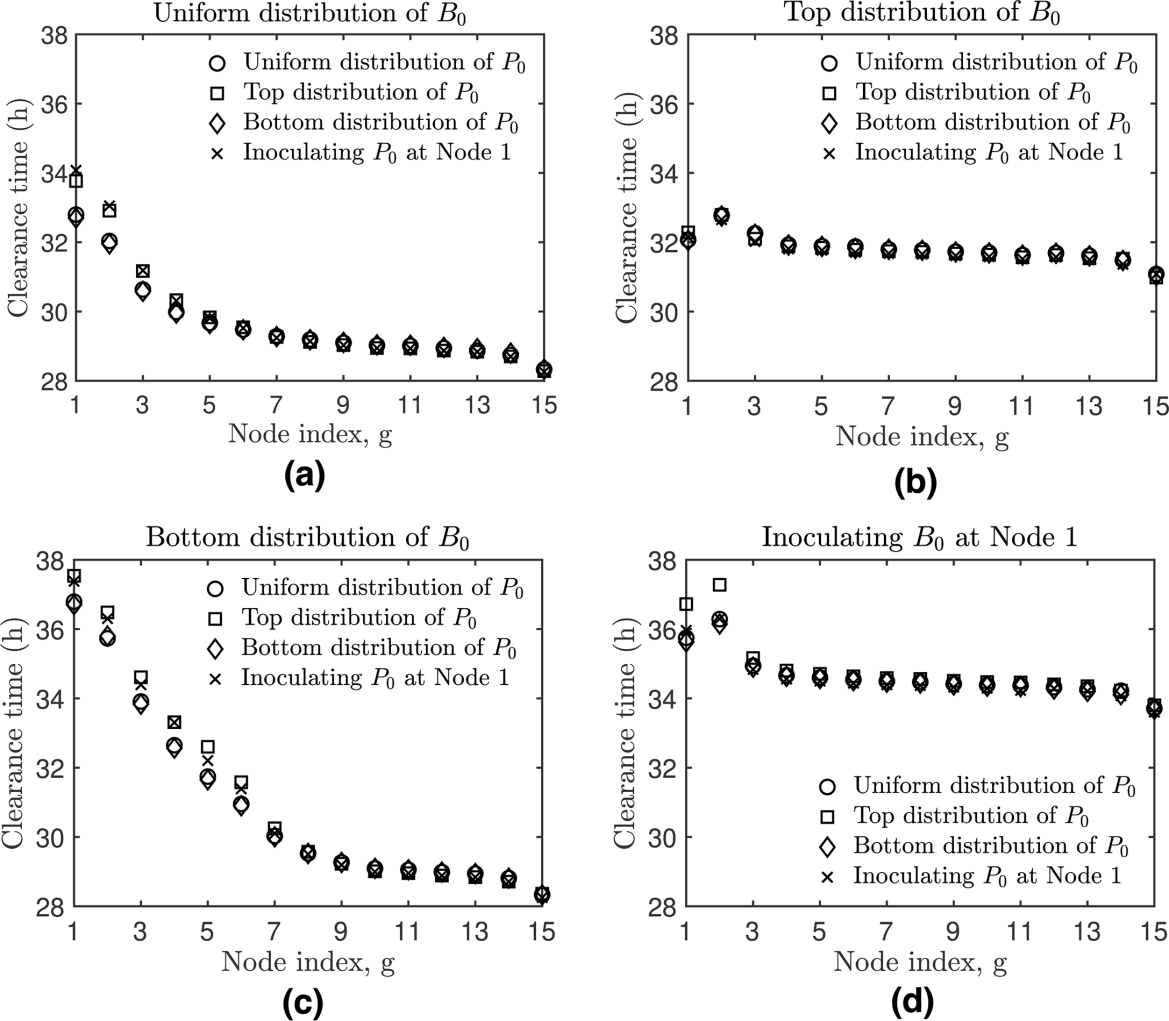
Infection elimination time given variations in the distribution of the phage dose and bacterial inoculum in the bronchial network. We evaluate different forms of allocating the bacterial inoculum ($B_{0}$) and the phage dose ($P_{0}$) among network nodes and calculate the infection clearance time at the node level (g). Distributions include (**a**) uniform distribution of the bacterial inoculum among all network nodes; (**b**) distribution of the bacterial inoculum between the first three nodes (**c**), among the last 12 nodes of the network, or (**d**) exclusively within the first node of the network. We use the same distribution forms as the bacterial inoculum for the phage dose (different markers inside plots). The simulation configuration is provided in the main text with parameter values in Table 1 and computational details in Text S1.

When the bacterial inoculum is uniformly distributed, the model predicted bacterial elimination occurred first at the bottom (28 hours) and later at the top nodes (32 to 34 hours) regardless of how the phage dose was allocated in the network (Fig. 4a). When the first three nodes were inoculated with bacteria, it took ∼4 hours to colonize the bottom nodes and around 32 hours to eliminate the infection from the network (Fig. S5, 2nd column from the left). In this case, the delay in the bacterial colonization of bottom nodes led to delayed infection dynamics and pathogen elimination in bottom nodes such that clearance times were more similar between bottom (31 hours) and top nodes (32 to 33 hours), regardless of initial phage allocation (Fig. 4b). When the bacterial inoculum was distributed among the last twelve nodes of the network, it took ~37 hours to clear the infection from the network. In this scenario, there was a longer infection clearance time difference between the top (clearance occurred at 37 hours) and the bottom (clearance occurred at 28 hours) of the network, attributed to a delay in bacteria colonizing the top of the network (Fig. 4c). Finally, the inoculation of bacteria in node 1 (trachea) resulted in the colonization of bottom nodes after ~6 hours and elimination of the infection after 36 hours (Fig. S5, last right-column); difference in infection elimination times was observed between the bottom (~34 hours) and the network’s top (~37 hours), Fig. 4d. Overall, we observed different bacterial colonization patterns depending on how the bacterial inoculum was initially distributed in the network (Fig. S5). Varying the allocation of the phage dose in the network did not significantly impact bacterial colonization or infection clearance patterns. As in previous simulations (Fig. 2 and 3e), we found a bottom-to-top pattern of infection resolution across different network distributions of phage and bacteria inocula. The pattern is compatible with the bacterial extinction threshold we set in our model based on the number of bacterial cells falling below the 1 CFU threshold and the node volume differences between the bottom and top nodes. For a detailed explanation of the factors contributing to this spatial pattern of infection elimination, refer to Text S1 D.

### Sufficient neutrophil levels and phage adsorption rates are required for effective clearance of *P. aeruginosa* lung infection

Next, we evaluated how intermediate phage efficacy and host innate immunity states impact metapopulation model outcomes, comparing the results with those from the well-mixed case. To model intermediate immune states, we vary the percentage of neutrophil availability in the lungs from 1% to 100%, where 100% availability corresponds to $∼3.24\times 10^{6}$ lung neutrophils in immunocompetent mice (31). To explore intermediate phage efficacy, we vary the phage adsorption rate across a range from $10^{-9}$ to $10^{-6}$ ${\left(ml/PFU\right)}^{\sigma }h^{-1}$. We simulate the phage treatment of a *P. aeruginosa* infection by inoculating a host with $10^{6}$ bacterial cells and introducing $10^{7}$ phage 2 hours after the bacterial infection. To assess the robustness of model predictions, we randomize the initial conditions and try 84 different ways of allocating the bacterial inoculum and the phage dose in the network. Then, we calculate the probability of clearing the infection by simulating the different initial conditions, given a specific immune state and phage adsorption rate.

**Fig 5:**
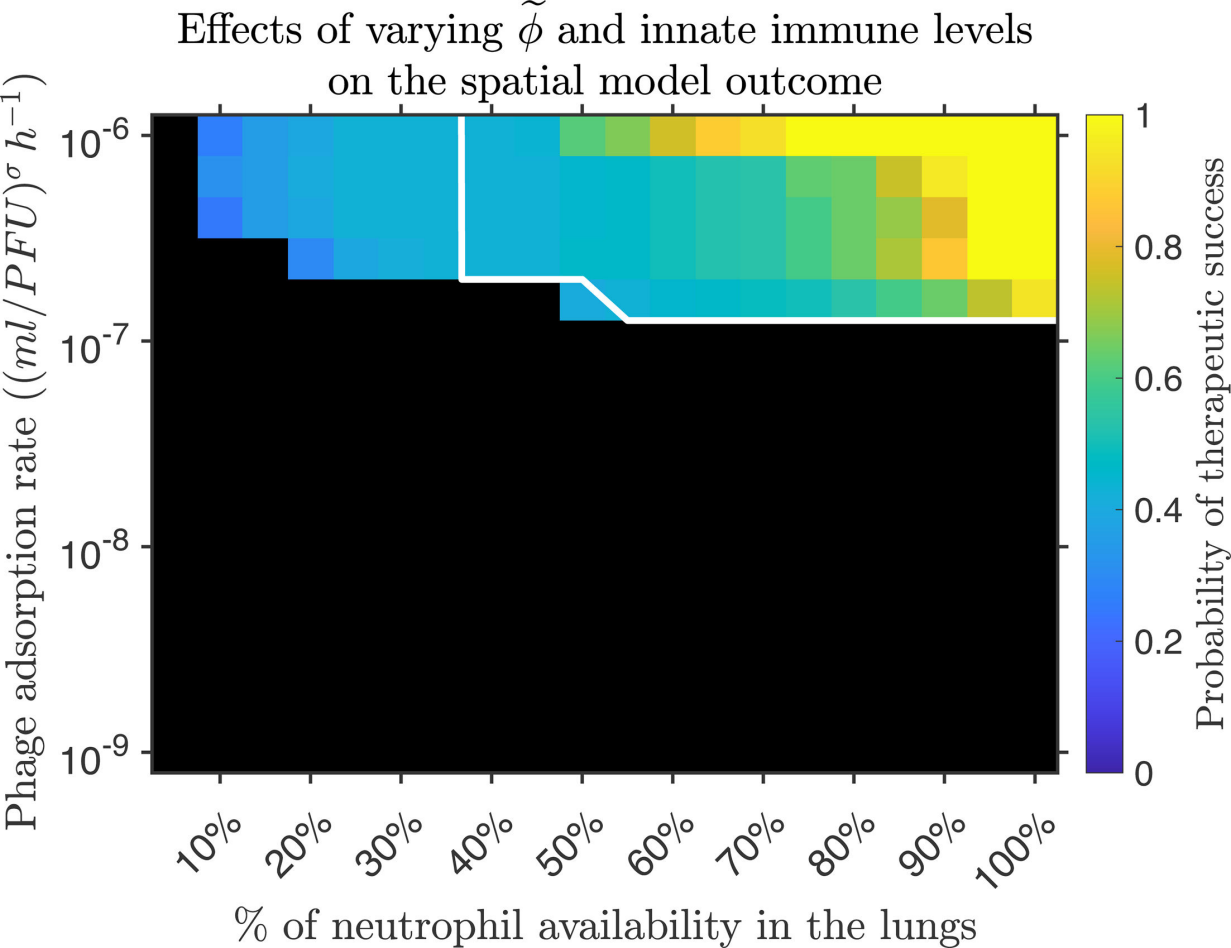
Probability of therapeutic success given intermediate phage efficacy and host innate immune levels. The probability of therapeutic success is examined given the variation in the percentage of neutrophils available (*x*-axis, from 1% to 100%) and phage adsorption rate (*y*-axis, from $10^{-9}$ to $10^{-6}$ ${\left(ml/PFU\right)}^{\sigma }h^{-1}$). Therapeutic success is measured based on 84 different initial distributions of the phage and bacterial inoculum. The colored regions represent a p>0 of clearing the infection, while black regions represent failure to clear the infection, i.e., a p=0 of therapeutic success. The white solid line contours the region of infection clearance predicted by the well-mixed model. The simulation configuration is provided in the main text with parameter values in Table 1 and computational details in Text S1. Here, mucin is set to a 2.5% level.

When neutrophil availability is limited (e.g., 10%–35%), the metapopulation model predicted therapeutic success ranging between 25% and 42% for phage adsorption rates ($\tilde{\phi }$) ranging between $∼2.5\times 10^{-7}$ and $10^{-6}$ ${\left(ml/PFU\right)}^{\sigma }h^{-1}$ (Fig. 5). In contrast, the well-mixed model, lacking consideration of the spatial structure, predicts infection persistence in the same parameter range. The spatial model predicts therapeutic success ranging between 40% and 100% when $\tilde{\phi }>10^{-7}$${\left(ml/PFU\right)}^{\sigma }h^{-1}$ and for neutrophil availability >50%. In contrast, infection was always resolved in the well-mixed model within the specified parameter range (Fig. 5, region limited by the solid white line). For example, if we compare the metapopulation model predictions for $\tilde{\phi }=1.58\times 10^{-7}$ ${\left(ml/PFU\right)}^{\sigma }h^{-1}$, neutrophil availability should be >85% to have increased chances of therapeutic success (i.e., >60%), while for the same $\tilde{\phi }$ value, infection always clears in the well-mixed model when neutrophil availability is >55%. These findings highlight the role of spatial structure in shaping phage therapy outcomes and suggest that higher levels of host innate immunity might be required to successfully clear a *P. aeruginosa* infection when spatial constraints are considered.

Within the metapopulation model outcomes, we noticed a trend where the higher the neutrophil availability, the wider the range of $\tilde{\phi }$ values for which the model predicts increased chances of infection clearance. This suggests that despite spatial structure effects that may affect phage–bacteria encounters and the phage adsorption rate, having a robust immune response facilitates the control of the *P. aeruginosa* infection. On the other hand, low phage efficacy, e.g., $\tilde{\phi }<10^{-7}$${\left(ml/PFU\right)}^{\sigma }h^{-1}$, may render phage therapy inefficient even when the host is fully immunocompetent, according to predictions from both the metapopulation and well-mixed models (Fig. 5, black region). We interpret these findings as follows. If phage efficacy is low, phage and bacteria can coexist even in the presence of the immune system. The conditions leading to coexistence depend on the relative balance between phage infection, immune killing, and bacterial growth, as demonstrated in previous theoretical work for a well-mixed model (19). According to the current metapopulation model, below a phage adsorption rate of $10^{-7}$ ${\left(ml/PFU\right)}^{\sigma }h^{-1}$, the infection cannot be cleared as phage and susceptible bacteria coexist, and eventually, bacteria reach the carrying capacity due to the proliferation of phage-resistant bacteria, even in the presence of a fully active immune response. This coexistence regime is depicted in Fig. S7, where we simulated our metapopulation model for a $\tilde{\phi }=9\times 10^{-8}$ ${\left(ml/PFU\right)}^{\sigma }h^{-1}$ just below the infection clearance transition, without allowing for phage resistance. Yet, phage, susceptible bacteria, and the innate immune system enter a coexistence state, and the infection is not cleared. Note that we also explored how variations in mucin and innate immune levels impact phage therapy outcomes. The exploratory analysis confirms the synergistic elimination of the infection by phage and neutrophils given sufficiently active immune states that are weakly impacted by mucin level variation (Fig. S8; Text S1 E).

### Analysis of *in vivo P. aeruginosa* infection data confirms a bottom-to-top infection clearance pattern

**Fig 6:**
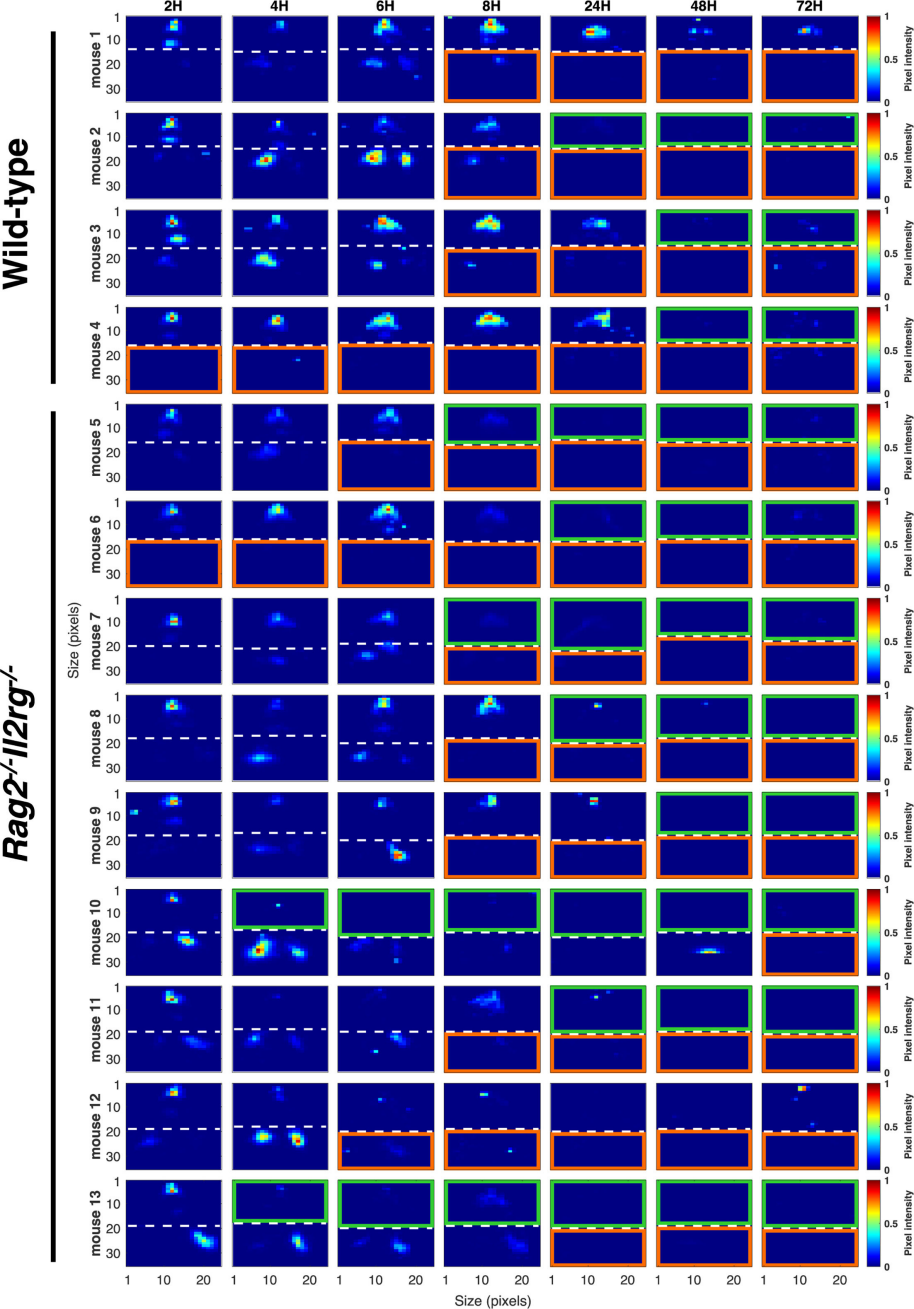
*In vivo P. aeruginosa* murine pneumonia data. Images depict infection dynamics of *P. aeruginosa* strain PAKlumi *in vivo* during 72 hours. Data are for two mice groups, WT (*N* = 4; mouse #1 to 4) and $Rag2^{-/-}Il2rg^{-/-}$ (*N* = 9; mouse #5 to 13). The bioluminescence signal represents the intensity of the infection in different mouse regions—a proxy for bacterial densities. A pixel intensity of 1 represents the highest bacterial density, while 0 represents the limit of detection. The white dashed line separates the upper and lower compartments of the mouse respiratory system. The orange and green boxes highlight the approximate time when the total intensity signal drops below the intensity threshold in the lower and upper compartments, respectively. Mice were inoculated with $10^{7}$ *P. aeruginosa* cells, and 2 hours after the bacterial inoculation, mice were treated with phage PAK_P1 ($10^{8}$ PFU).

**Fig 7:**
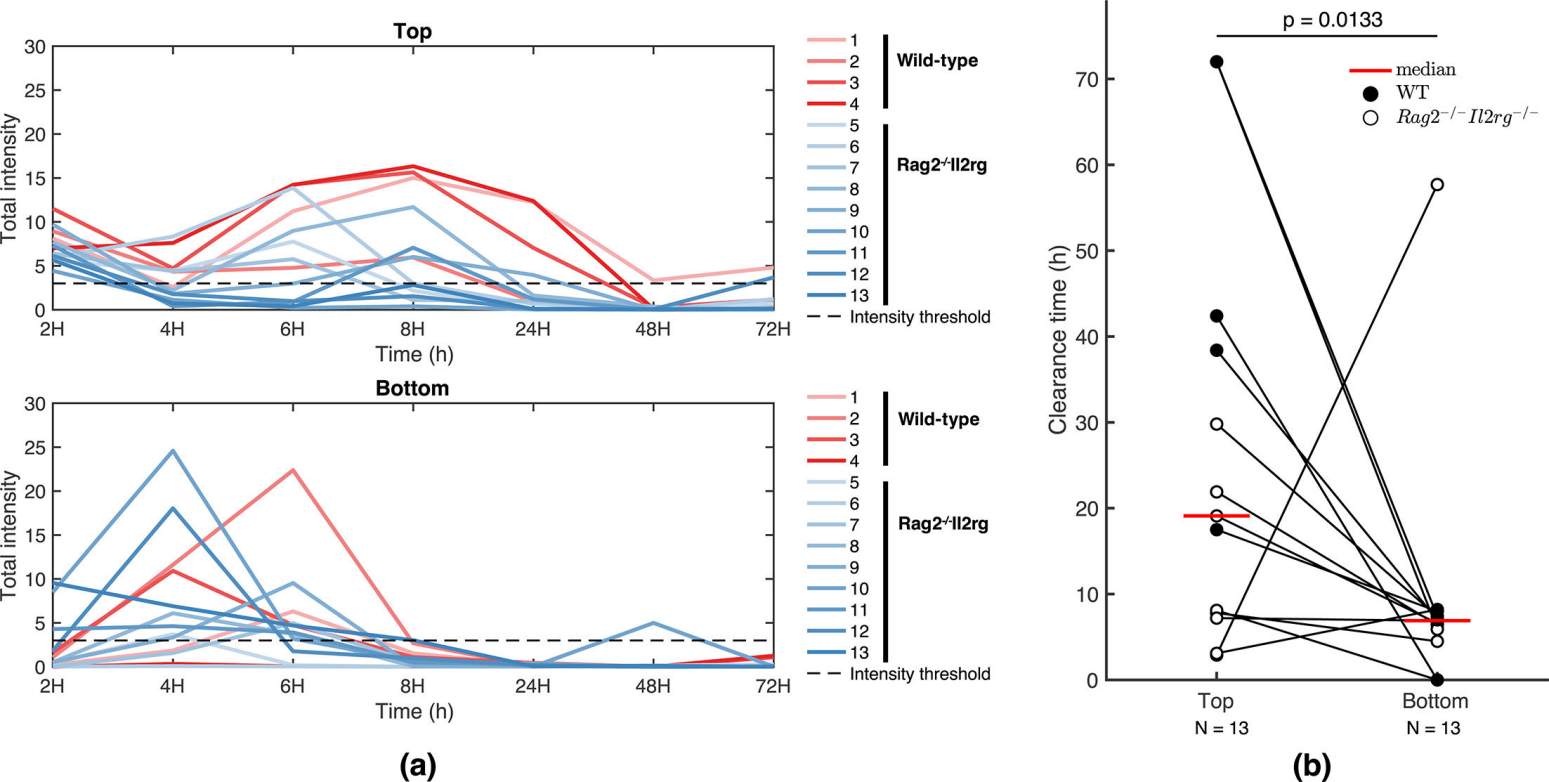
Time series of the total intensity signal and the infection clearance analysis using *in vivo P. aeruginosa* murine pneumonia data. We show the time series of total intensity signals for the upper and lower compartments of 13 mice (**a**). The total intensity of one compartment is calculated by adding the pixel intensity values from all pixels making up a compartment. The black dashed line represents the intensity threshold (value of 3) below which the total intensity signal clears. We calculate the time to infection resolution for the upper and lower compartments (**b**). We use data from 13 mice, including WT (*N* = 4) and $Rag2^{-/-}Il2rg^{-/-}$ (*N* = 9) mice groups. We used the one-sided Wilcoxon signed rank test to compare the infection clearance time difference between the upper and lower compartments (**b**).

Our finding of a systematic bottom-to-top transition pattern supported by multiple simulations prompted us to examine the spatial patterns of clearance within *in vivo* imaging data from phage treatment of *P. aeruginosa-*infected mice. We analyzed the bioluminescence infection signal from images (Fig. 6) generated in a previous phage therapy study (13). The data set included images of 13 mice, comprising both wild-type (WT) (*N* = 4) and lymphocyte-deficient $Rag2^{-/-}Il2rg^{-/-}$ (*N* = 9) mice groups. Following *P. aeruginosa* strain PAKlumi infection ($10^{7}$ CFU), mice were treated with phage PAK_P1 ($10^{8}$ PFU) 2 hours later. We split individual mouse images into two compartments (dashed white line in Fig. 6). The top compartment encompassed the nose and throat, while the bottom compartment included the lungs. To quantify infection intensity within each compartment, we computed the total intensity signal by summing the pixel intensity values of all pixels within a compartment (Fig. 7a). This total intensity served as a proxy for the bacterial infection status inside the mouse, where higher values indicated higher bacterial density. To determine infection signal clearance in each compartment, we established an intensity threshold of 3, below which we considered the total intensity signal as cleared. In Text S1 F, we include comprehensive details on the image analysis methodology.

Using the time series of imaged mice, we tracked the progression of the infection in the two compartments (Fig. 7a). We observed a more rapid decrease in the intensity signal below the threshold in the bottom compartment (∼6–8 hours after the onset of infection) compared to the top compartment (∼8–48 hours after the onset of infection). The observation implies a faster clearance of infection in the lower respiratory tract than in the upper respiratory tract of mice. Upon calculating the infection clearance time for both compartments, we found that the intensity signal cleared faster in the bottom compartment than in the top compartment. Specifically, the median time to infection clearance was 7 hours for the bottom compartment and 19 hours for the top compartment, yielding a statistically significant difference with a *P*-value of 0.0133 (Fig. 7b). This spatial pattern of infection resolution qualitatively aligns with predictions made by the metapopulation model, especially under conditions involving active innate immunity and phage therapy.

## DISCUSSION

In this study, we developed a metapopulation model of phage therapy of a *P. aeruginosa* lung infection. We modeled the ecological interactions between phage, two *P. aeruginosa* strains (phage-susceptible and phage-resistant), and the host innate immune response at the airway level. The dynamics arising from the phage therapy of a *P. aeruginosa* infection in an immunocompetent host indicates that phage therapy successfully eliminates bacterial infections when the host innate immune responses complement phage treatment. The therapeutic outcome was robust to heterogeneous distributions of the phage dose and bacterial inoculum in the network and to variations in mucin levels. However, limited neutrophil availability and a low phage adsorption rate may negatively impact phage treatment efficacy. Moreover, the metapopulation model required higher innate immune response levels to increase the likelihood of therapeutic success, compared to a well-mixed model that achieved infection resolution at lower innate immune levels, highlighting the role of spatial structure in shaping phage therapy outcomes. Across our model simulations, we observed a spatial pattern of infection clearance, with infection resolving faster in the bottom nodes compared to the top nodes. This pattern arises partly due to the dynamics crossing a critical, lower threshold via a volumetric effect influenced by the smaller number of bacterial cells per airway in the bottom vs top nodes. Finally, by analyzing *in vivo* data of mice infected with *P. aeruginosa* and treated with phage, we observed that bacterial infections clear faster in the lower than the upper respiratory tract. This result is qualitatively similar to the bottom-to-top infection elimination pattern predicted by the model. We note that the imaging data for these mice are coarse-grained, preventing us from compartmentalizing the images at an airway-level resolution corresponding to the model scale. Therefore, the mismatch in resolution between the data and the model simulations allows only for a qualitative comparison of the spatiotemporal patterns. Despite this limitation, we found a similar directionality in pathogen elimination in both the data and the model under comparable conditions of immune and phage effects.

The therapeutic outcome recapitulated in our model is consistent with *in vivo* phage therapy results, where phage efficiently controlled respiratory bacterial infections in both humans (41–43) and mice (13, 44–46). Notably, we found that the therapeutic outcome was robust to different distributions of the phage dose across the bronchial network, including heterogeneous and homogeneous dose distributions. This is consistent with outcomes from phage delivery strategies using phage-loaded microparticles to distribute phages throughout the lung, effectively reducing infections caused by *P. aeruginosa (47*) and *S. aureus (48*). Furthermore, Delattre *et al*. developed a computational model that recapitulated phage–bacteria interactions *in vivo* and found that varying the route of administration of the phage dose, i.e., intratracheally or intravenously, did not impact the therapeutic outcomes with both courses producing similar effects (44). While variations in bacterial inoculum allocation impacted colonization patterns within the network, they did not disrupt the therapeutic outcome. These collective findings suggest that therapeutic success might be robust to initial phage and bacterial distributions in the lungs, though quantitative differences can arise with mismatched distributions.

Immunophage synergy does have its limits, whether *in vivo (13*), in mean field models (19), or as shown here, in metapopulation models. For example, a weak innate immune response may negatively impact phage treatment efficacy. Our model predicted a minimum of 45% of neutrophil availability to start clearing bacteria from the lungs, although higher innate immunity levels are needed to increase the likelihood of therapeutic success. This is consistent with *in vivo* experiments showing that phage therapy failed to control a *P. aeruginosa* infection when mice had an impaired innate immune system, e.g., when mice were either MyD88-deficient (13) or neutropenic (13, 14). On the other hand, phage therapy successfully eliminated the infection when mice were immunocompetent (13, 14, 45). Future phage therapy studies and clinical trials should examine the importance of the host immune status in determining treatment efficacy.

The metapopulation network model of phage–bacteria–immune dynamics has caveats. For example, the network structure of the model is based on a symmetrical bronchial tree, while the mouse bronchial tree is asymmetric (49). Hence, future model extensions could consider modeling asymmetric branching structures and their effects on phage and bacteria propagation across the bronchial tree. By doing so, we can evaluate if tree asymmetries facilitate spatial refuges for bacteria to protect themselves from phage attacks. A critical defense mechanism of the respiratory system is mucociliary clearance (MCC) (50), where motile cilia of the airway epithelium transport a mucus layer out of the lungs, carrying particles trapped in the mucus layer. Currently, our model does not address the (stochastic) mechanism by which bacteria first infect the lungs, the mucosal layer’s involvement in this process, or the potential translocation of bacteria between different body sites (51). In the future, we could extend the model to include MCC effects to assess how MCC impacts bacterial colonization, infection clearance patterns, and neutrophil transport within the airways. Our model does not account for the interactions between pathogenic *P. aeruginosa* and the lung microbiome (52). Furthermore, we do not consider the effects of other innate effector cells, such as macrophages, the predominant immune cells in the mice lungs before infection begins (31). The role of alveolar macrophages in the activation of neutrophils, phagocytosis, and the possible interactions these cells have with phage (53, 54) should be considered for future model extensions.

Phage therapy has emerged as an alternative to chemical antibiotics to treat infections by MDR bacteria. However, spatial structure effects of *in vivo* environments where phage and bacteria interact have not been fully addressed. Here, we used a metapopulation network model to provide further support that synergistic interactions between phage and neutrophils can lead to bacterial elimination within the spatially structured lung environment. However, higher innate immune levels could be required to increase the likelihood of therapeutic success in a spatially structured environment compared to a well-mixed system. Our modeling outcomes further demonstrated the importance of the host immune status and phage life history traits, such as phage adsorption rate, in shaping the therapeutic outcomes. In the future, it will be important to obtain high-resolution *in vivo* temporal data and exploit it to achieve fully quantitative model-data comparisons. Such advances will be key to improving the predictive power of *in vivo* phage therapy models at finer scales. The emergence of bottom-to-top spatiotemporal infection clearance patterns also suggests that extending *in vitro* phage therapy models may help guide therapeutic treatment development in other *in vivo* contexts.

## MATERIALS AND METHODS

### Parameter estimation

The parameter values used in the simulations of the metapopulation model are shown in Table 1. Much of the parameter estimation was carried out in previous work (see the “Parameter Estimation” section in reference 13). Airway anatomical information, including airway length and diameter, was obtained from (28). Bacteria and phage diffusion constants can be found in Table S1.

### Model simulation

Model equations (equations 1-4) are numerically integrated using ODE45 in MATLAB R2020b. By doing so, we obtain the population dynamics of phage-susceptible and phage-resistant bacteria, phage, and the innate immune response. We assume bacteria become extinct in a node when the number of bacteria drops below the extinction threshold of 1 CFU. This applies to both $B_{S}$ and $B_{R}$. When bacterial counts drop below the extinction threshold in a node, we set the bacterial density to 0 CFU/mL.

## Data Availability

The code and data used to simulate the metapopulation model, perform the image analysis, and generate the main figures, as well as the supplemental figures, can be found in the GitHub repository at https://github.com/RogerRln/metapop_lung.

## SUPPLEMENTAL MATERIAL

Supplemental material msystems.00171-24-s0001.pdf Supplemental text, table, and figures.

ASM does not own the copyrights to Supplemental Material that may be linked to, or accessed through, an article. The authors have granted ASM a non-exclusive, world-wide license to publish the Supplemental Material files. Please contact the corresponding author directly for reuse.
