# Supplementary material for: Metapopulation model of phage therapy of an acute Pseudomonas aeruginosa lung infection
Source: mSystems. 2024 Sep 4;9(10):e00171-24. doi: 10.1128/msystems.00171-24 (PMC11562898; doi:10.1128/msystems.00171-24)
Supplement: Supplemental material — Supplemental text, table, and figures. [file msystems.00171-24-s0001.pdf]

## Supplementary Text S1: Model details

### A. Determining the number of generations ($g$) of the metapopulation network based on total mouse's lung volume

We determine the number of generations ($N$) that the metapopulation network must have, based on the volume of the network ($V_{network}$) and the total lung capacity (TLC) of mice[1], which is approximately 1 ml. To do so, we consider the volume of individual airways ($V_{airway,g}$) and the number of airways present at each generation, $\sim 2^{g-1}$. Then, we find $N$ by approximating $V_{network}$ to the TLC of the mouse.

$$V_{network} \approx \sum_{g=1}^{N} V_{airway,g} \times 2^{g-1} \approx 1\, ml. \tag{S11}$$

We find that with $N = 15$, we do not exceed the TLC of the mouse, and we obtain a network volume of ∼0.9 ml. We use 15 generations (nodes) for the metapopulation network model.

### B. Calculating bacteria and phage diffusion constants as a function of mucin level

The hopping rate is calculated as the reciprocal of the time it would take for bacteria and phage to cross half of the airway via diffusion, $D$. Hence, we need to determine the diffusion constants of phage and bacteria to calculate their hopping rate. The evidence shows that the diffusion constant of phage[2,3] and the speed of bacteria[4] are shaped by the concentration of the mucus lining the airways. For example, highly concentrated mucus can reduce bacteria motility and slow phage diffusion. Our model focuses on mucin levels that are physiologically relevant in acute lung infections (e.g., 0-4% mucin concentration). Below, we explain how we calculate the diffusion coefficients of phage and bacteria across different mucin levels.

We collected speed values of *P. aeruginosa* for two different mucin levels[4] (2.5% and 8%). We use the speed values to calculate the diffusion coefficient of bacteria using the run-and-tumble model as a proxy,

$$D_B = \frac{v^2 t}{3(1-\alpha_B)} = \frac{v^2 t}{2}. \tag{S12}$$

Where $v$ is the bacterial speed in $\mu m/s$, $t = 1\, s$, is the duration of the bacterial runs, and $\theta \sim 60$ degrees, is the average change in direction between runs, such that $\alpha_B = \langle \cos(> \theta) \rangle \approx 1/3$.

To find additional bacteria speed values within our target range of mucin concentrations (0-4%), we fit a line between the data points we collected (Fig. S1a). By calculating the parameters of the linear function, we can find the speed of bacteria for different mucin levels relevant to our biological system. Then, we calculate the bacteria diffusion coefficient from the speed value using the run-and-tumble model (Eqn. S12).

We calculate the phage diffusion constant across different mucin levels by using the Stokes-Einstein equation (Eqn. S13). To do so, we use the predicted values of mucus viscosity ($\eta_M$) across different mucin levels using the formula, $\eta_M = a \cdot [mucin]^b + \eta_W$, obtained from[3]. In that study, they fitted the mucus viscosity to empirical data using two power functions for two different regions of mucin levels, low [0-1% mucin] and high [1-4% mucin] concentrations. The parameter $a$ has values of 0.326 and 0.323 in the low and high mucin regions, respectively. The exponent $b$ has values of 0.65 and 1.59 for the low and high mucin regions, respectively. Parameters $a$ and $b$ can also be found in Tables 3 and 4 of the study[3].

$$D_P = \frac{kT}{6\pi \eta_M R_P}. \tag{S13}$$

For the phage diffusion constant calculations, thermal energy was assumed at 37°C such that $kT = 4.28\, pN \cdot nm$ and water viscosity was $\eta_W = 0.69\, mPa \cdot s$. The phage radius ($R_P$) was calculated to be 90 $nm$, half the length of a typical T4 phage[3]. Using the Stokes-Einstein equation (Eqn. S13) and the predicted values of mucus viscosity across different mucin levels, we can calculate the phage diffusion constant for different mucin levels (Fig. S1b).

Using the relationship between mucin levels and phage and bacteria diffusion coefficients, we can explore how lung physiological conditions impact mucus concentration and affect the infection dynamics. For example, variations in the concentration of mucin could impact the time it takes for bacteria to colonize different nodes at various depths of the bronchial tree, phage dispersion times, the establishment of phage resistance, and the clearance of the infection by the combined effects of phage and neutrophils at different depths of the bronchial tree.

### C. Calculating the hopping rate of bacteria and phage

| Bacteria and phage diffusion constants and their hopping rates | | | | | |
|---|---|---|---|---|---|
| Species | Diffusion in $\mu m^2 s^{-1}$ (feasible range) | Diffusion in $cm^2 h^{-1}$ (feasible range) | Example: time ($\tau$) to cross half of the trachea of length, $l$ = 0.76 cm | Hopping rate ($h^{-1}$) | Ref |
| Bacteria ($D_B$) | 144 (295-80) | 0.0052 (0.01 - 0.003) | $\tau_B$ = 13.88 h | $\frac{1}{13.88h} = 0.072\,h^{-1}$ | 4–7 |
| Phage ($D_P$) | 1.21 (3.7 - 0.7) | $4.35 \times 10^{-5}$ ($1.33 \times 10^{-4} - 2.52 \times 10^{-5}$) | $\tau_P$ = 1659 h | $\frac{1}{1659} = 6.02 \times 10^{-4}\,h^{-1}$ | 2,3,8 |

TABLE S1: Examples of phage and bacteria diffusion coefficients under normal mucus concentration (2.5%). In parentheses, we show a feasible range of diffusion values when mucin levels vary from 0 to 4%.

We calculate the hopping rate of bacteria and phage using the airway length information[9] and the diffusion constants of phage and bacteria (Table S1). The hopping rate is calculated as the reciprocal of the time, $\tau$, it would take for each species to cross half of the airway, $l/2$, via diffusion, $D$. We leverage the relationship between the mean squared displacement (MSD) and 1D Brownian motion, $\langle (x - x_0)^2 \rangle = 2Dt$, to calculate $\tau_B$ and $\tau_P$.

$$\tau_B = \left(\frac{l}{2}\right)^2 \frac{1}{2D_B} = \frac{l^2}{4}\frac{1}{2D_B} = \frac{l^2}{8D_B}, \tag{S14}$$

$$\tau_P = \left(\frac{l}{2}\right)^2 \frac{1}{2D_P} = \frac{l^2}{4}\frac{1}{2D_P} = \frac{l^2}{8D_P}. \tag{S15}$$

Then, we take the reciprocal of $\tau_B$ and $\tau_P$ to calculate the hopping rates of bacteria and phage, respectively. The hopping rate determines how fast bacteria and phage leave a local airway and hop to a neighboring airway. We use the hopping rates to calculate the influx and outflux terms of equations 1-3.

$$\frac{1}{\tau_B} = \frac{8D_B}{l^2}, \tag{S16}$$

$$\frac{1}{\tau_P} = \frac{8D_P}{l^2}. \tag{S17}$$

### D. Spatial pattern of infection elimination, innate immunity effects

The synergistic clearance of *P. aeruginosa* infection by phage and host innate immunity yielded a recurring feature: a spatial pattern of clearance of pathogens from bottom-to-top of the bronchial network. This pattern is compatible with a situation in which phage initially help decrease bacterial density to the point where the immune system alone can control and drive bacteria to extinction. Once bacteria fall below a critical level ($K_D$), the immune system removes bacteria at a fixed rate until complete elimination within each node. We hypothesize that the observed difference in clearance times between the bottom and top nodes is influenced, in part, by node volume differences. Our model associates the bacterial extinction threshold with the number of bacterial cells crossing the 1 CFU threshold at the node level, suggesting that the spatial pattern in pathogen clearance could be a result of dynamics crossing a critical threshold via a volumetric effect associated with the smaller total number of bacterial cells in bottom vs. top nodes. If this were the case, the clearance time at the node level could be predicted using the immune killing rate ($\varepsilon K_I$), providing insights into the spatial variations observed in the metapopulation model simulations.

When calculating clearance time, we make several assumptions, including that $B_S$ is the predominant bacterial type throughout the simulation, that bacterial density levels are below a critical threshold ($B_S << K_D$) at final simulation times, and that host innate immunity reached its maximum level ($K_I$) by the end of the simulation. These conditions have been met in prior simulations (Fig. 2) depicting phage therapy for a *P. aeruginosa* infection in an immunocompetent host. Moreover, it is important to note that low bacterial density levels ($B_S << K_C$) enable positive exponential growth ($r_P B_S$), and such growth should be considered when calculating the infection clearance time.

To determine the time to bacterial extinction, we solve the $\frac{dB_S}{dt}$ equation describing how $B_S$ population changes over time due to the innate immune response and exponential growth. After solving the equation, we isolate $t$ to determine the clearance time.

$$\frac{dB_S}{dt} = r_P B_S - \varepsilon K_I B_S, \tag{S18}$$

$$\int \frac{dB_S}{B_S} = (r_P - \varepsilon K_I) \int dt, \tag{S19}$$

$$\ln(B_S) = r_P t - \varepsilon K_I t + \ln(B_{S0}), \tag{S110}$$

$$t = \frac{\ln(B_{S0}) - \ln(B_S)}{\varepsilon K_I - r_P}. \tag{S111}$$

Bacteria become extinct in node $i$ once their levels fall below the $1/V_i$ density threshold, where $V_i$ is the volume of node $i$. So, the time to bacterial extinction, denoted as $t_i$, for node $i$ is

$$t_i = \frac{\ln(B_{S0}) - \ln(\frac{1}{V_i})}{\varepsilon K_I - r_P}, \tag{S112}$$

$$t_i = \frac{\ln(B_{S0}) + \ln(V_i)}{\varepsilon K_I - r_P}. \tag{S113}$$

We note that time to infection resolution in node $i$ is proportional to the natural logarithm of the node volume ($V_i$) and scales with the inverse of the immune killing rate adjusted by the per capita bacterial growth rate ($r_P$).

To further characterize the infection clearance time (ICT) difference between nodes, we calculate the ICT difference between node $i$ and the last network node (which is the node with the smallest volume), i.e., $t_i - t_{bottom}$. To do so, we use the clearance time calculated in Eqn. S113. The ICT difference between nodes due to the immune killing rate is,

$$t_i - t_{bottom} = \frac{1}{\varepsilon K_I - r_P} \times \ln\left(\frac{V_i}{V_{bottom}}\right). \tag{S114}$$

We observe that the clearance time difference is proportional to the natural logarithm of the volume ratio, $V_i/V_{bottom}$, where $V_{bottom}$ is the volume of the last network node. We conclude that a larger volume difference between nodes corresponds to a longer clearance time difference. The largest volume difference in the network occurs between node one (trachea) and node fifteen (terminal airway), resulting in the longest ICT difference between any pair of nodes. Comparing the theoretical ICT difference to that obtained from simulations, we note that the theoretical ICT difference aligns with the simulation results (Fig. S6, purple line). This result highlights that, as phage drive bacteria below a critical level, the immune system effectively controls and drives bacteria to extinction.

### E. Evaluating the impact of variations in realistic mucin levels and intermediate innate immune states on the phage therapeutic outcome

As an additional model analysis, we explore how variations in mucin and innate immune levels impact phage therapy outcomes. To model intermediate immune states, we vary the percentage of neutrophil availability in the lungs from 1% to 100%, where 100% availability corresponds to $\sim 3.24 \times 10^6$ lung neutrophils in immunocompetent mice[10]. Then, we vary mucin levels across a physiologically relevant range for acute lung infections, ranging from 0% to 4%. Moreover, we use the phage adsorption rate, $\widetilde{\phi} = 1.686 \times 10^{-7}$ (ml/PFU)$^{\sigma} h^{-1}$, for our model simulations. To simulate the phage treatment of a *P. aeruginosa* infection, a host is inoculated with $10^6$ bacterial cells, and after 2 hr, the host is treated with $10^7$ phage. To assess the robustness of model predictions, we randomize the initial conditions and try 84 different ways of allocating the bacterial inoculum and the phage dose in the network. Then, we calculate the probability of clearing the infection by simulating the different initial conditions, given a specific mucin level and innate immune state.

When neutrophil availability is >45%, the metapopulation model predicted a ∼43% probability of clearing the infection regardless of mucin level (Fig. S8). The result contrasts with the prediction of the well-mixed model, where infection always clears when ≥45% of lung neutrophils are available (Fig. S9). Increasing neutrophil availability to 80% increases the chances of therapeutic success to 66%, especially for low mucin levels ranging from 0% to 2% (Fig. S8). The higher probability of clearing the infection in low mucin levels suggests that when metapopulation dynamics are more homogeneous, the elimination of bacteria by phage and neutrophils is facilitated. Phage and bacteria spread

faster in low mucin levels, so their population dynamics homogenize among network nodes. Consequently, neutrophil resources are homogeneously distributed in the network, easing infection control. On the other hand, high mucin levels limit the diffusion of both phage and bacteria, causing them to predominantly occupy nodes proximal to their inoculation sites. This results in heterogeneous population dynamics and uneven distribution of neutrophil resources across network nodes. We hypothesize that limited neutrophil resources and high mucin levels may negatively impact the phage therapeutic outcome.

The model predicted a 100% chance of eliminating the infection when the host is fully immunocompetent (i.e., 100% neutrophil availability) and mucin levels vary between 0-3% (Fig. S8). This outcome is consistent with previous simulations, where we tested different distributions of phage dose and bacterial inoculum in a fully immunocompetent host (Fig. S5). Overall, outcomes suggest that the metapopulation spatial structure influences the dynamics of the infection and the phage therapeutic outcome.

### F. Analysis of *in vivo* imaging of *P. aeruginosa* infected mice

We use images of mice generated previously in an *in vivo* phage therapy study[11] to extract the luminescence signal indicating the presence of a bacterial infection. In that study, authors used a *P. aeruginosa* PAKlumi strain to infect several groups of mice and the *P. aeruginosa* phage PAK_P1 to treat the infected animals. They track the evolution of the infection for 72 hours, taking pictures at 2, 4, 6, 8, 24, 48, and 72 hours post-infection using the IVIS imaging system. We focus on a group of 14 mice, including immunocompetent wild-type (N=4) and lymphocyte deficient $Rag2^{-/-}Il2rg^{-/-}$ (N=10) mice, as they tend to survive the bacterial infection when treated with phage. Both WT and $Rag2^{-/-}Il2rg^{-/-}$ mice groups produce neutrophils during the bacterial infection.

The IVIS imaging system acquires a photographic image of the animal and overlays the bioluminescent infection signal on the image[12] to create a composite picture of the infected animal. We use MATLAB R2020b and its image-processing capabilities to read and extract data from the time series of imaged mice. Using the images of mice, we select a region of interest (24 pixels width x 35 pixels height) that covers the animal's lungs, throat, and nose. Then, we select a border that separates the upper and the lower compartments of the mouse respiratory system. For instance, the upper compartment includes the nose and throat, while the mouse lungs are in the lower compartment. After splitting the region of interest into two compartments, we can analyze the progression of bacterial infection in each compartment.

We pre-process the images containing bioluminescence signals and normalize the pixel intensity values of all images to a 0-1 scale. Pixel intensity 1 represents a high bacterial density, while 0 represents no signal of detected bacterial infection. We extract the infection signal from a particular compartment by adding the pixel intensity values of all the pixels that make up that compartment. We called this value the total intensity signal. We evaluate the progress of the bacterial infection in both the upper and lower compartments by examining the changes in the total intensity signal over time.

### G. Calculating infection clearance time from *in vivo* mice infection data

We are interested in calculating the time to infection resolution using the images of infected mice and comparing it with the clearance time predicted by the metapopulation model. To do so, we define an intensity threshold below which the total intensity signal is cleared and assume this corresponds to infection clearance. Inspection of images of infected mice and differences in intensity of both upper and lower compartments enabled the selection of an infection threshold of 3.

We define some filters to consider which mice to use in the infection clearance analysis. For example, when a mouse dies before 72 hr, i.e., before the completion of the experiment, we discard that mouse for our analysis. After filtering out our dataset, we kept four immunocompetent wild-type and nine lymphocytes deficient $Rag2^{-/-}Il2rg^{-/-}$ mice for a total N = 13 mice.

We calculate the time to infection resolution as the time it takes for the total intensity signal to fall below the intensity threshold (conditional on the signal never exceeding the intensity threshold again after the signal falls below the threshold). When the total intensity signal of one compartment does not exceed the intensity threshold during the experiment (72 hr), we set the time to infection resolution to 0 hr for that compartment. We calculate the time to infection resolution for both upper and lower compartments. Finally, we compare the clearance time difference between the compartments.

### H. Statistical analysis

Statistical analyses were conducted in MATLAB R2020b. The one-sided Wilcoxon signed-rank test was used to compare the time to infection resolution between the upper and lower compartments of the mouse. $p < 0.05$ was considered statistically significant.

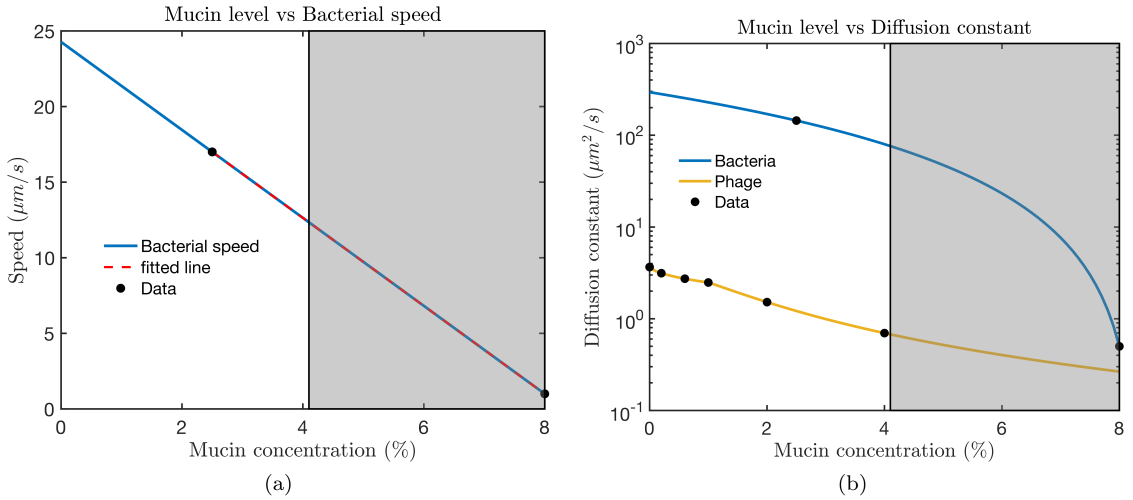


FIG. S1: **Relationship between mucin concentration and bacterial speed and phage diffusion**. Bacteria speed values were collected from[4] for two mucin levels, 2.5 and 8% (a). We fit a line (red dashed line) between the data points to find additional speed values for intermediate mucin levels (e.g., 0-4% mucin). In (b), we depict the relationship between mucin level and bacteria and phage diffusion. The gray boxes show high mucin levels not used in our model simulations.

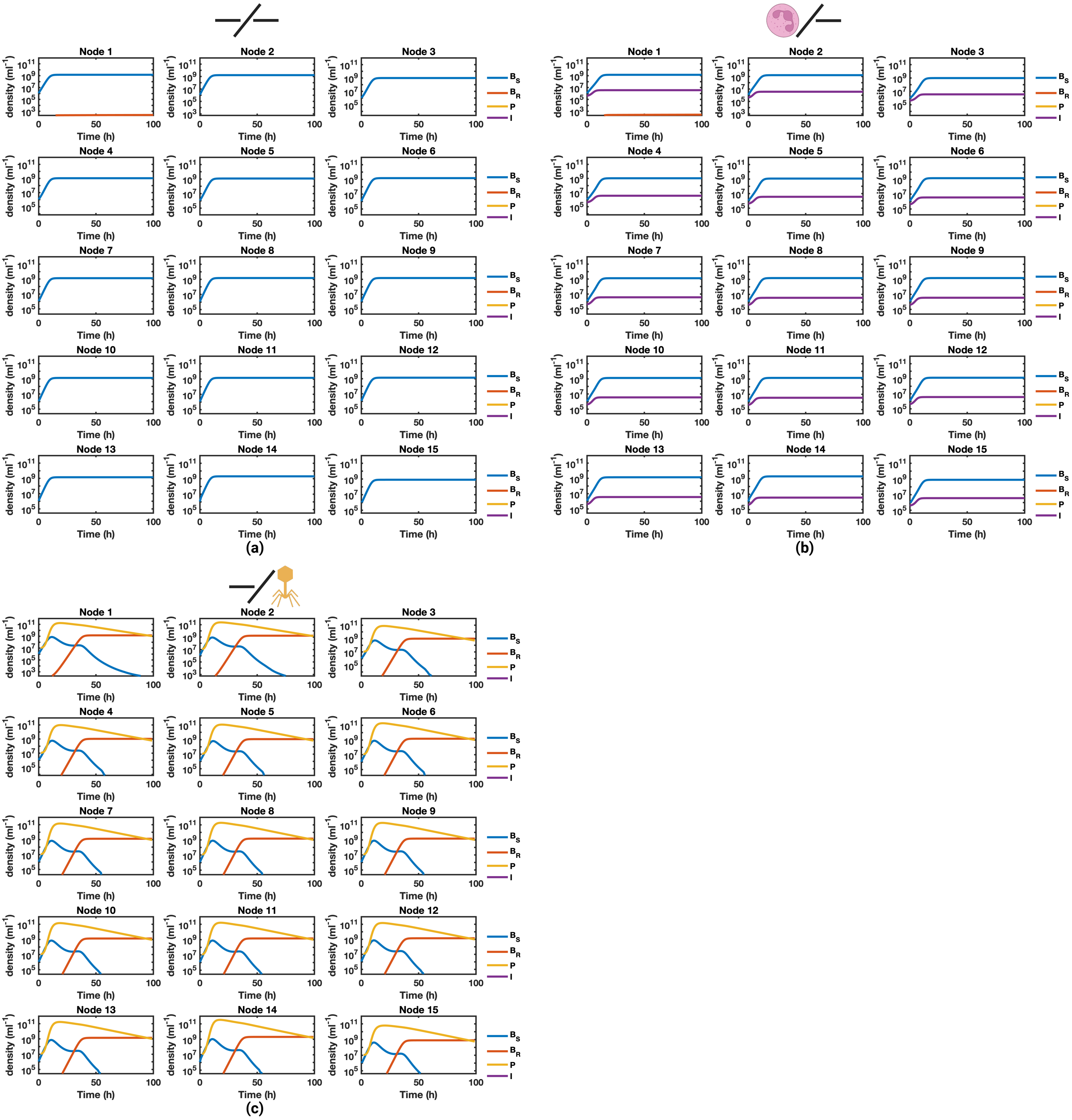


FIG. S2: **Population dynamics at the node level under different phage and immune treatments**. We show the dynamics of phage (solid yellow line), phage-susceptible bacteria (solid blue line), phage-resistant bacteria (solid orange line), and the host innate immune response (purple solid line) as a result of no treatment (a), innate immune treatment (b), and phage therapy (c). We infect a host with $10^6$ bacterial cells. When used, phage ($10^7$ PFU) are administered 2 hr after the bacterial infection. When the host is immunocompetent (b), we set the initial immune density to $I_0 = 4.05 \times 10^5$ cells/ml in all the network nodes. We uniformly distribute the phage dose (c) and the bacterial inoculum (a-c) in the network such that each node had the same initial bacterial density ($1.11 \times 10^6$ CFU/ml) and phage density ($1.11 \times 10^7$ PFU/ml). The simulation runs for 100 hr. Here, Node 1 = Generation 1 = trachea, and Node 15 = Generation 15 = terminal airway.

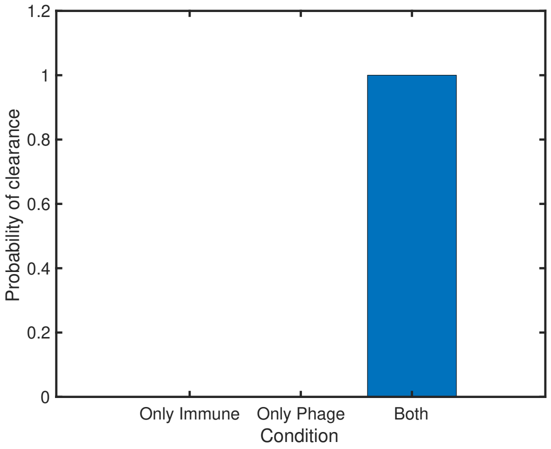


(a)

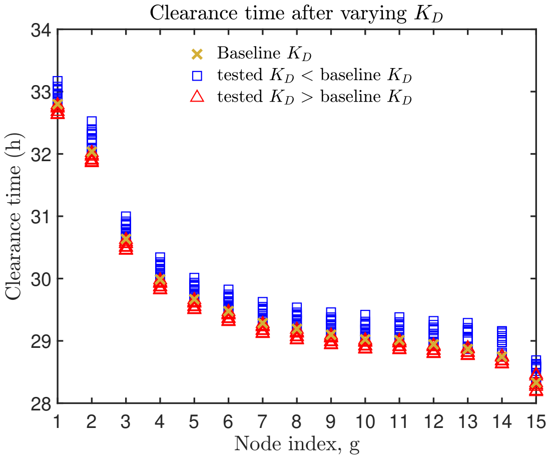


(b)

FIG. S3: **Probability of infection elimination and infection clearance time given variations in parameter** $K_D$. We randomly draw 20 values, allowing them to vary within ±20% of the original value of $K_D$ ($6.14 \times 10^6$ CFU/ml), and calculate the probability of eliminating the infection under different treatment conditions, including only innate immunity, only phage therapy, or the combination of active innate immunity and phage therapy (a). We then show the infection elimination times at the node level for the condition of active innate immunity + phage therapy (b). A gold cross marker indicates the clearance time for the original value of $K_D$. We initiate the infection by inoculating a host with $10^6$ bacterial cells. If phage therapy is used, we administer $10^7$ phage (PFU) 2 hr after the beginning of the infection. When the host innate immunity is active, we set an initial immune density of $4.05 \times 10^5$ cells/ml in all nodes.

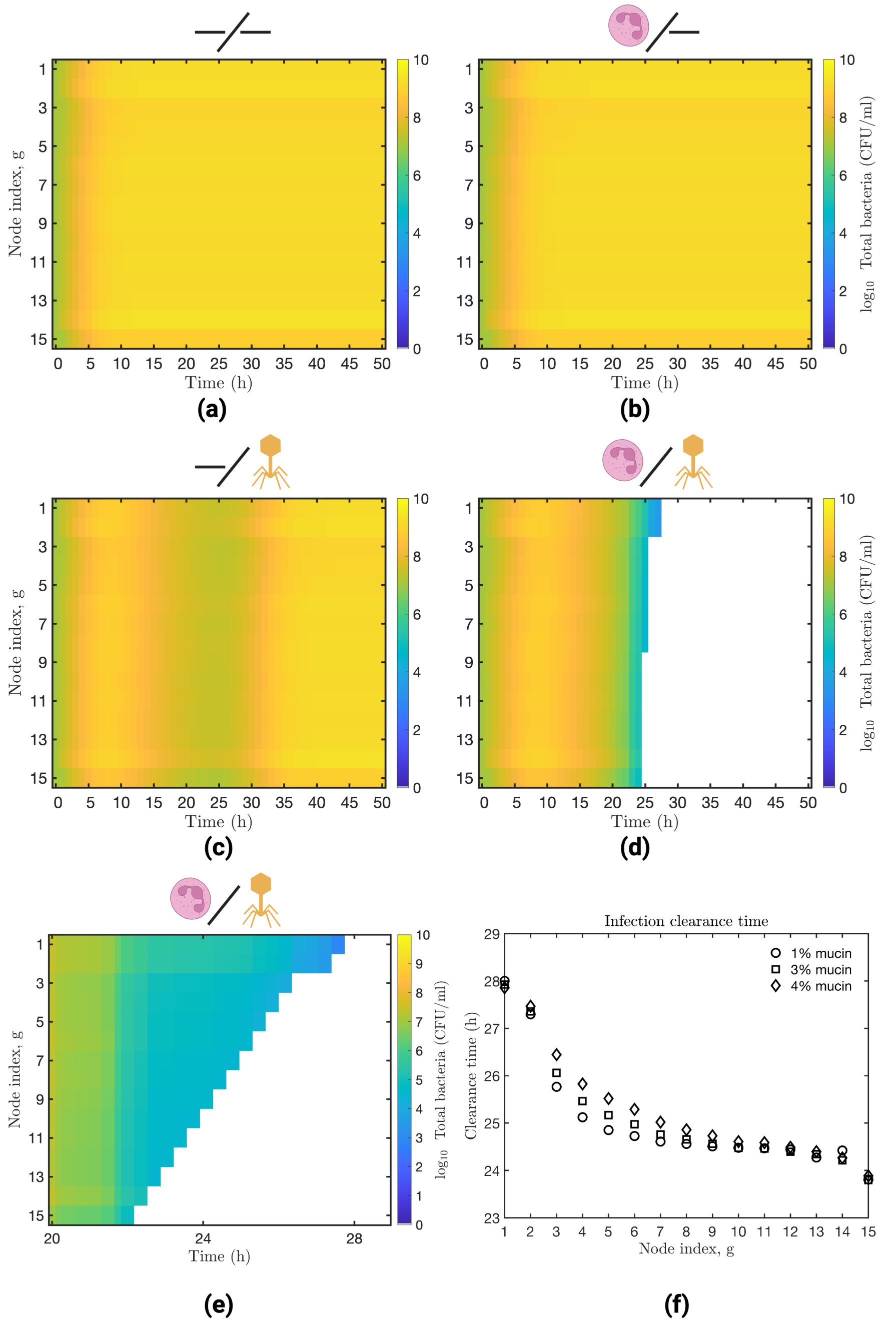


FIG. S4: **Bacterial dynamics given high initial phage and bacterial inocula**. We simulate four treatment scenarios that result from the presence or absence (−) of both phage and the innate immune response. We show the bacterial dynamics across the metapopulation network when the host is immunodeficient untreated (a) or phage-treated (c). Similarly, we show the bacterial dynamics when the host is immunocompetent untreated (b) or phage-treated (d). The heatmaps depict the progression of the bacterial infection across the network; each row represents a network node, $g$, while the columns indicate the simulation time (hr). The node color represents the bacterial density at a given time. The yellow regions represent high bacterial density, and the white areas represent infection clearance. When the host is immunocompetent and phage-treated, we zoom in and show the infection clearance pattern (e). We also test the effects of varying the mucin level (1-4%) on the infection clearance time (f). We inoculate a host with $10^7$ bacterial cells. If phage therapy is used, we administer $10^8$ phage (PFU) 2 hr after the beginning of the infection. We uniformly distribute the phage dose and the bacterial inoculum among network nodes. When the host is immunocompetent, we set an initial immune density of $4.05 \times 10^5$ cells/ml in all the nodes. If the host is immunodeficient, we set the immune density to $I_0 = 0$ cells/ml. A 2.5% mucin concentration was used for scenarios (a) to (e). All the simulations ran for 50 hr.

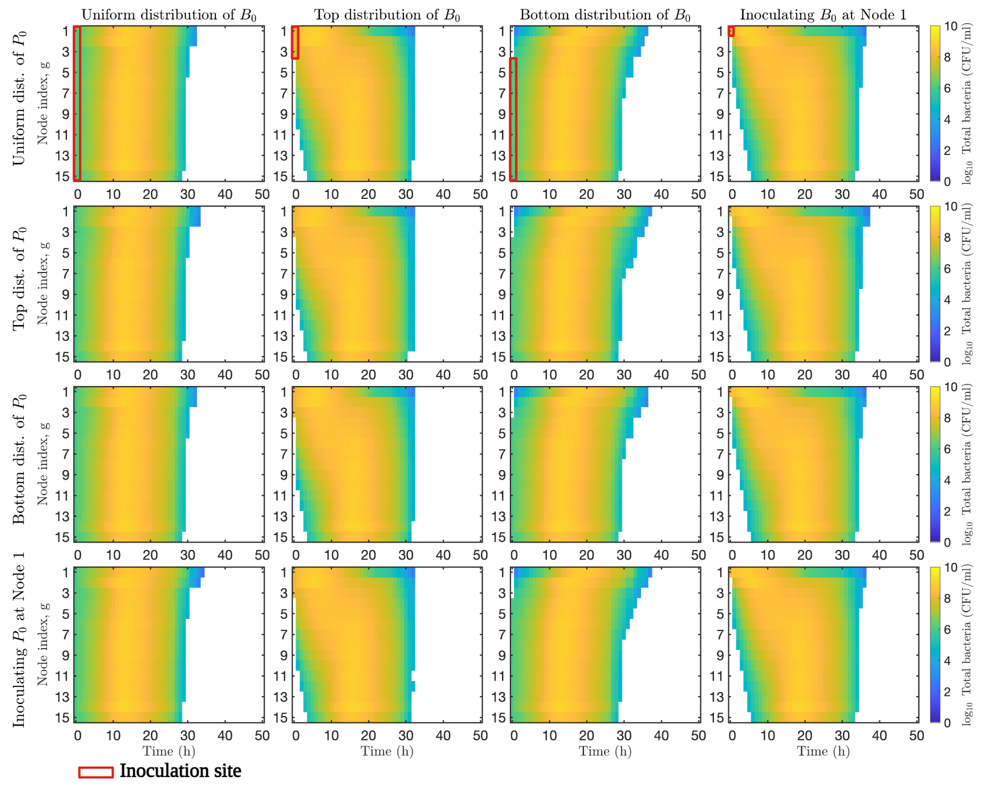


FIG. S5: **Infection dynamics given variations in the distribution of phage dose and bacterial inoculum in the bronchial network**. We evaluate different forms of allocating the bacterial inoculum ($B_0$) and the phage dose ($P_0$) among network nodes. For example, 1) we uniformly distribute the phage dose or the bacterial inoculum among the network nodes (1st-left column), 2) we distribute the phage dose or the bacterial inoculum between the first three nodes of the network (Top distribution, 2nd-left column), 3) we distribute the phage dose or the bacterial inoculum among the last 12 nodes of the network (Bottom distribution, 3rd column), or 4) we inoculate the first node of the network with phage or bacteria (4th column). We use a heatmap to represent infection dynamics resulting from paired distributions of phage dose and bacterial inocula. We show how bacterial infection progresses per network node ($g$). In the heatmap, each row represents a network node, while the columns indicate the simulation time. The node color represents the bacterial density at a given time, the yellow regions represent high bacterial density, and the white areas represent infection elimination. We infect an immunocompetent host with $10^6$ bacterial cells. We administer $10^7$ phage (PFU) 2 hr after the bacterial infection. We set an initial immune density of $I_0 = 4.05 \times 10^5$ cells/ml in all the nodes. The simulation ran for 50 hr.

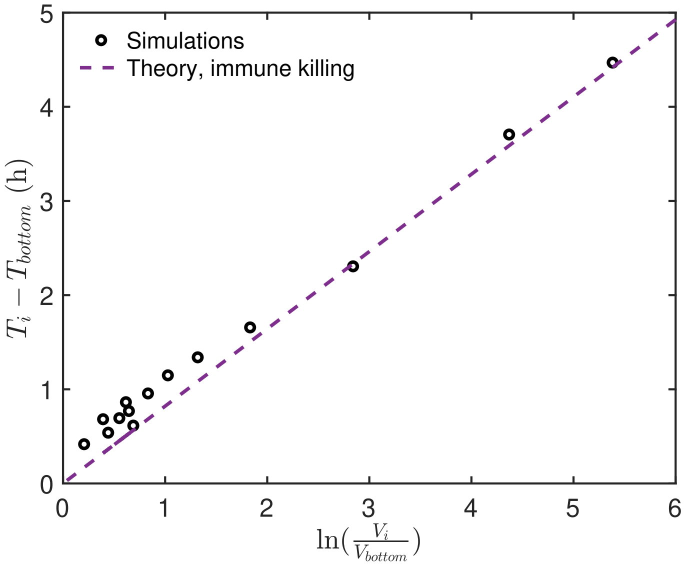


FIG. S6: **Infection clearance time difference across the network, theory vs simulations**. We show the infection clearance time difference between node $i$ and the last node of the network due to the immune killing, comparing theory (dashed purple line) vs simulations (open circles). The theoretical time difference due to immune killing was previously calculated in Eq. S114. Model simulations are based on Fig. 2, where we simulate the phage treatment of a *P. aeruginosa* infection in an immunocompetent host. There, the phage dose and the bacterial inoculum were uniformly distributed such that each node had the same initial bacterial density ($1.11 \times 10^6$ CFU/ml) and phage density ($1.11 \times 10^7$ PFU/ml). The initial immune density was set to $I_0 = 4.05 \times 10^5$ cells/ml in all the nodes.

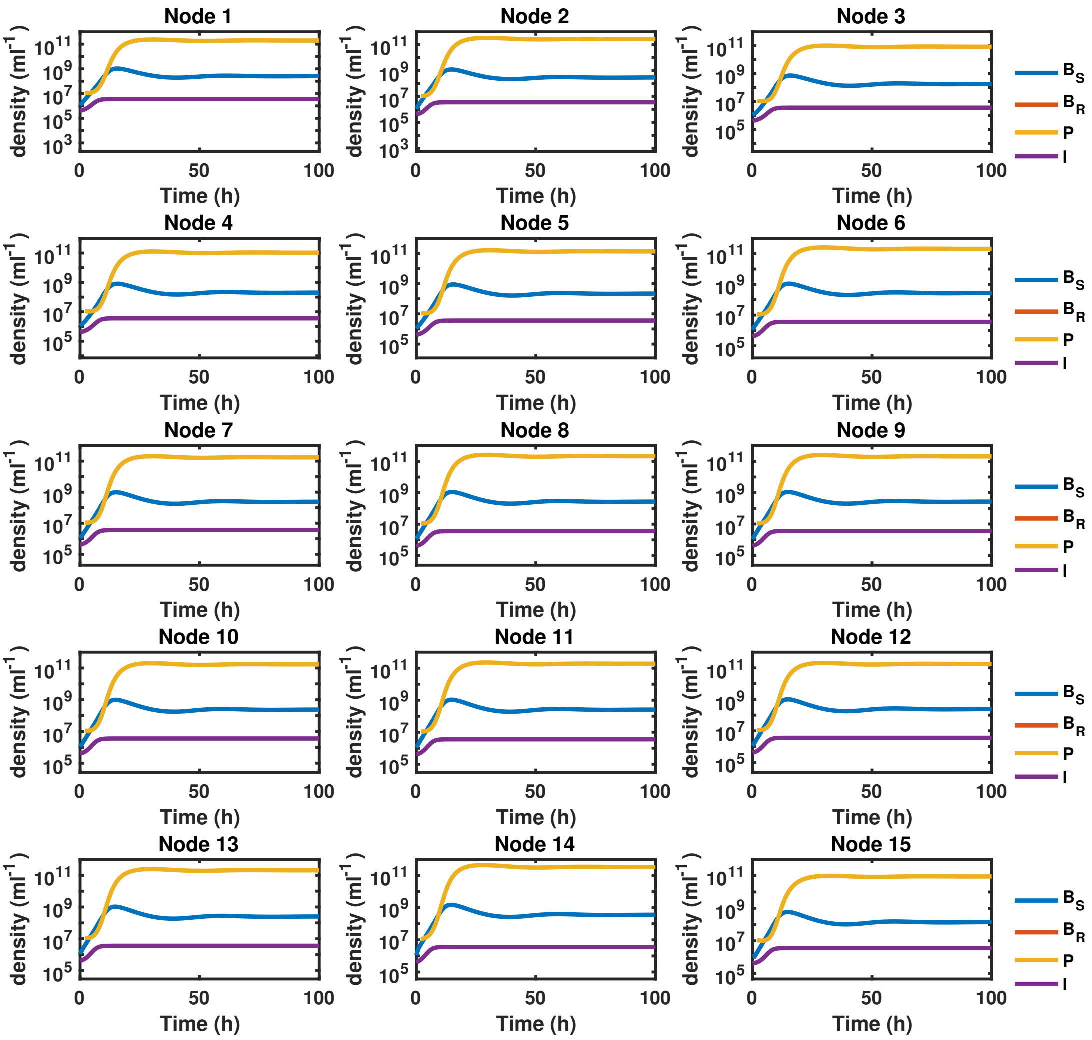

FIG. S7: **Population dynamics for the coexistence regime**. We show the dynamics of phage (solid yellow line), phage-susceptible bacteria (solid blue line), and the host innate immune response (purple solid line). We inoculated an immunocompetent host with $10^6$ bacterial cells and administered $10^7$ PFU 2 hr later. We set the initial immune density to $I_0 = 4.05 \times 10^5$ cells/ml in all the network nodes. We uniformly distribute the phage dose and the bacterial inoculum among network nodes. To establish the phage-bacteria coexistence regime we use $\widetilde{\phi} = 9 \times 10^{-8}$ (ml/PFU)$^\sigma h^{-1}$ and to block the emergence of phage resistance we set $\mu_1 = 0$. The simulation runs for 100 hr. Here, Node 1 = Generation 1 = trachea, and Node 15 = Generation 15 = terminal airway.

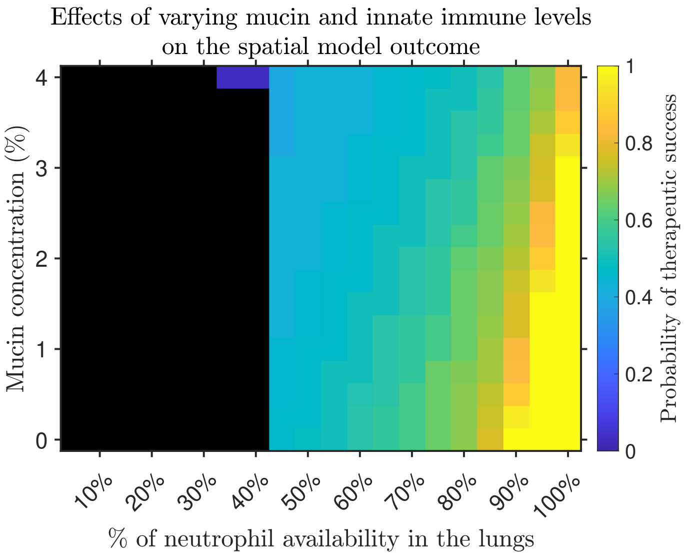


FIG. S8: **Probability of therapeutic success given intermediate mucin levels and innate immune states**. To explore intermediate innate immune responses, we vary the percentage of neutrophils available in the lungs (1-100%). Further, we vary the mucin levels within a range (0-4%) that is physiologically relevant for acute lung infections. To simulate the phage treatment of a *P. aeruginosa* infection, we inoculate a host with $10^6$ bacterial cells and introduce $10^7$ phage 2 hr after the bacterial inoculation. We calculate the probability of clearing the infection by simulating 84 different initial conditions given a specific innate immune state and mucin concentration. The heatmap shows the probability of clearing the infection. The colored regions represent a $p > 0$ of clearing the infection, while black regions represent a $p = 0$ of therapeutic success. The simulation runs for 250 hr. A 100% neutrophil availability represents $\sim 3.24 \times 10^6$ lung neutrophils in an immunocompetent mouse[10]. For the simulations, we use a phage adsorption rate value of $\widetilde{\phi} = 1.686 \times 10^{-7}$ (ml/PFU)$^\sigma h^{-1}$.

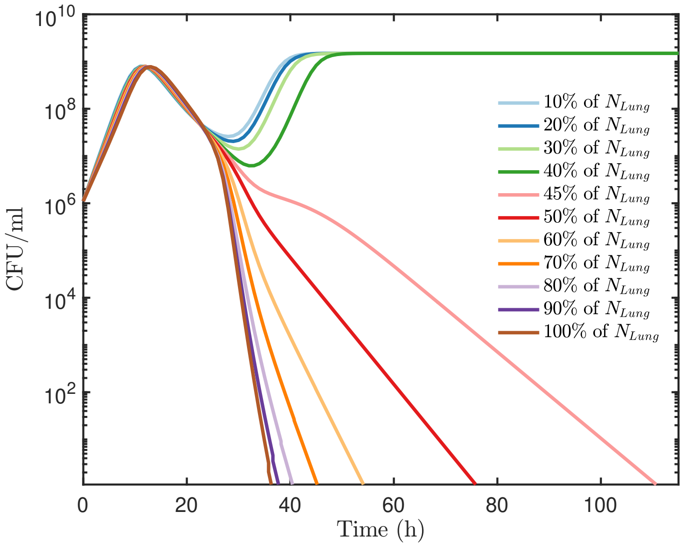


FIG. S9: **Bacterial dynamics of the well-mixed model for intermediate innate immune states**. We show total bacterial dynamics ($B_{tot} = B_S + B_R$) that result from infecting a host with $10^6$ *P. aeruginosa* cells. Phage therapy ($10^7$ PFU) is administered 2 hr after the bacterial inoculation. To model intermediate immune response levels, we vary the percentage of neutrophils available in the lungs from 10 to 100%. We consider a total lung volume of 0.9 ml. The simulation runs for 115 hr. A 100% neutrophil availability represents $\sim 3.24 \times 10^6$ lung neutrophils in an immunocompetent mouse[10].

[1] C. G. Irvin and J. H. Bates, "Measuring the lung function in the mouse: the challenge of size," *Respiratory research*, vol. 4, pp. 1–9, 2003.

[2] J. J. Barr, R. Auro, N. Sam-Soon, S. Kassegne, G. Peters, N. Bonilla, M. Hatay, S. Mourtada, B. Bailey, M. Youle, *et al.*, "Subdiffusive motion of bacteriophage in mucosal surfaces increases the frequency of bacterial encounters," *Proceedings of the National Academy of Sciences*, vol. 112, no. 44, pp. 13675–13680, 2015.

[3] K. L. Joiner, A. Baljon, J. Barr, F. Rohwer, and A. Luque, "Impact of bacteria motility in the encounter rates with bacteriophage in mucus," *Scientific reports*, vol. 9, no. 1, pp. 1–12, 2019.

[4] H. Matsui, V. E. Wagner, D. B. Hill, U. E. Schwab, T. D. Rogers, B. Button, R. M. Taylor, R. Superfine, M. Rubinstein, B. H. Iglewski, *et al.*, "A physical linkage between cystic fibrosis airway surface dehydration and pseudomonas aeruginosa biofilms," *Proceedings of the National Academy of Sciences*, vol. 103, no. 48, pp. 18131–18136, 2006.

[5] M. Wu, J. W. Roberts, S. Kim, D. L. Koch, and M. P. DeLisa, "Collective bacterial dynamics revealed using a three-dimensional population-scale defocused particle tracking technique," *Appl. Environ. Microbiol.*, vol. 72, no. 7, pp. 4987–4994, 2006.

[6] B. R. Phillips, J. A. Quinn, and H. Goldfine, "Random motility of swimming bacteria: single cells compared to cell populations," *AIChE journal*, vol. 40, no. 2, pp. 334–348, 1994.

[7] M. Theves, J. Taktikos, V. Zaburdaev, H. Stark, and C. Beta, "A bacterial swimmer with two alternating speeds of propagation," *Biophysical journal*, vol. 105, no. 8, pp. 1915–1924, 2013.

[8] R. Moldovan, E. Chapman-McQuiston, and X. Wu, "On kinetics of phage adsorption," *Biophysical journal*, vol. 93, no. 1, pp. 303–315, 2007.

[9] W. B. Counter, I. Q. Wang, T. H. Farncombe, and N. R. Labiris, "Airway and pulmonary vascular measurements using contrast-enhanced micro-ct in rodents," *American Journal of Physiology-Lung Cellular and Molecular Physiology*, vol. 304, no. 12, pp. L831–L843, 2013.

[10] J. Reutershan, A. Basit, E. V. Galkina, and K. Ley, "Sequential Recruitment of Neutrophils into Lung and Bronchoalveolar Lavage Fluid in LPS-Induced Acute Lung Injury," *American Journal of Physiology-Lung Cellular and Molecular Physiology*, vol. 289, no. 5, pp. L807–L815, 2005.

[11] D. R. Roach, C. Y. Leung, M. Henry, E. Morello, D. Singh, J. P. D. Santo, J. S. Weitz, and L. Debarbieux, "Synergy between the Host Immune System and Bacteriophage Is Essential for Successful Phage Therapy against an Acute Respiratory Pathogen," *Cell Host & Microbe*, vol. 22, no. 1, pp. 38–47, 2017.

[12] E. Lim, K. D. Modi, and J. Kim, "In vivo bioluminescent imaging of mammary tumors using ivis spectrum," *JoVE (Journal of Visualized Experiments)*, no. 26, p. e1210, 2009.
